# Discovery of a blood-based miRNA signature that can predict onset of active tuberculosis among household contacts of TB patients

**DOI:** 10.3389/ftubr.2024.1415346

**Published:** 2024-10-29

**Authors:** Evangeline Ann Daniel, Kannan Thiruvengadam, Padmapriyadarsini Chandrasekaran, Nancy Hilda, Pavithra Umashankar, Pooja Prashanthi, Murugesan Selvachithiram, Sathyamurthi Pattabiraman, Brindha Bhanu, Amsaveni Sivaprakasam, Mandar Paradkar, Vandana Kulkarni, Rajesh Karyakarte, Shri Vijay Bala Yogendra Shivakumar, Vidya Mave, Amita Gupta, Luke Elizabeth Hanna

**Affiliations:** ^1^National Institute for Research in Tuberculosis, Indian Council of Medical Research (ICMR), Chennai, India; ^2^University of Madras, Chennai, India; ^3^BJ Government Medical College-Johns Hopkins Clinical Research Site, Pune, India; ^4^Johns Hopkins Center for Infectious Diseases in India, Pune, India; ^5^Byramjee Jeejeebhoy Government Medical College and Sassoon General Hospitals, Pune, India; ^6^Johns Hopkins University School of Medicine, Baltimore, MD, United States

**Keywords:** miRNA, tuberculosis, progression, QuantiFERON supernatant, biomarker

## Abstract

**Background:**

Non-sputum based predictive biomarkers capable of identifying individuals with high risk of progression to active tuberculosis (TB) are critical for global TB control. MicroRNAs (miRNAs) are significant regulators involved in TB pathogenesis and hence we aimed to identify a miRNA signature capable of predicting progression to TB disease.

**Methods:**

We compared the differential miRNA expression profile of QuantiFERON supernatants of TB Progressors, defined as healthy household contacts (HHCs) of TB patients, who developed active TB disease during a 2-year follow-up period, and Non-progressors defined as HHCs from the same longitudinal cohort who did not develop TB disease during the entire follow-up period, using the nanostring nCounter platform. Receiver Operator Characteristic (ROC) analysis was performed to evaluate the diagnostic accuracy of the identified miRNA biomarkers, followed by random forest analysis to determine the best predictive model.

**Results:**

We identified 30 differentially regulated miRNAs between the two groups. Of these, hsa-miR-585-3p and hsa-miR-92a-3p were up-regulated with a maximum fold change of 1.74 and 1.71 respectively, while hsa-miR-223-3p and hsa-miR-451a were down-regulated by −2.05 and −2.04 fold respectively. Random forest analysis revealed that hsa-miR-181a-5p, hsa-miR-204-5p, hsa-miR-197-3p, hsa-miR-92a-3p, hsa-miR-451a, hsa-miR-24-3p, and hsa-miR-487a-3p exhibited 100% accuracy in identifying Progressors. This panel of 7 miRNAs demonstrated excellent diagnostic performance characteristics with 100% sensitivity and specificity.

**Conclusion:**

We propose that the identified miRNA signature has the potential to serve as a very useful tool for early identification of individuals who bear the highest risk of progression to TB, so that they can be targeted for timely intervention.

## 1 Introduction

Tuberculosis (TB) remains one of the leading causes of death from a single infectious agent. Although one-fourth of the world's population is infected with *Mycobacterium tuberculosis* (*Mtb*), only about 5%−10% will go on to develop active TB, while the rest will continue to harbor *Mtb* in a controlled state, referred to as Latent Tuberculosis Infection (LTBI) (1). Reduced notification and missed diagnosis during the recent COVID-19 pandemic (2) has contributed to an enriched pool of latent TB reservoirs that constitute a potential source of new cases. The poor positive predictive value (PPV) of the currently available diagnostic tests for LTBI [*viz*. the tuberculin skin test (TST) and interferon gamma release assay (IGRA)] results in high numbers-needed-to-treat (NNT) making it extremely challenging to cater preventive therapy to the large LTBI pool, particularly in TB endemic countries, and in most cases unnecessarily (3). This calls for the identification of easily detectable non-sputum-based biomarkers that can precisely identify individuals with high risk of progression to TB disease with an optimal sensitivity and specificity of ≥90% or minimum sensitivity and specificity of ≥75% as per the Target Product Profile (TPP) proposed by World Health Organization (4).

The immune response to TB is multifaceted and highly complex. Small, non-coding RNAs called microRNAs (miRNAs) have emerged as critical regulators in orchestrating the TB disease pathogenesis (5). It is evident from recent studies that many of the immune responses elicited by the host, and the immune modulation strategies effectuated by *Mtb* to subvert them, are facilitated by miRNAs (6, 7). The differential expression pattern of circulating miRNAs in healthy, latent and active TB cases highlights their potential to serve as useful biomarkers (8, 9). Added to this, the remarkable stability and ease of detection of miRNAs in body fluids make them amenable to translation as point-of-care (PoC) tests.

In this study, we proposed to identify miRNA biomarkers/biosignatures that can predict risk of progression from *Mtb* infection to active disease. Considering that biomarkers based on antigen-specific responses enhance the specificity of a biomarker-based test, we for the first time used *Mtb* antigen stimulated QuantiFERON (QFT) supernatants for miRNA profiling.

## 2 Material and methods

### 2.1 Ethical approval

The study was conducted with the approval of the Institutional Ethics Committees of ICMR-National Institute for Research in Tuberculosis (ICMR-NIRT), Chennai, India, Byramjee Jeejeebhoy Government Medical College (BJGMC), Pune, India, and Johns Hopkins University (JHU), Baltimore, MD, United States. The Ethical approval number of the study is 2020021.

### 2.2 Study subjects

A cohort of HHCs of newly diagnosed pulmonary TB (PTB) patients was established at two sites, ICMR-NIRT and BJGMC, India, in collaboration with JHU, USA, as part of the Cohort for Tuberculosis Research by the Indo-US Medical Partnership (C-TRIUMPH) study (10), and the enrolled participants were followed up for approximately 2 years during August 2014 to December 2017. The definitions used for classifying study participants are provided in Table 1. All HHCs underwent clinical and laboratory assessment for TB at baseline and during each follow-up visit. TST and IGRA were performed at baseline and repeated at every visit if the previous test was negative. A HHC was confirmed to have active TB disease if he/she became positive for TB by culture or GeneXpert/MTB Rif during follow-up.

**Table 1:** Definitions used for classifying study participants.

**Classification**	**Definition**
Household contacts (HHCs)	Adults and children residing in the same house with a TB patient during 3 months before TB diagnosis in the index case.
Progressors	HHCs who developed TB at any time after 2 months of TB diagnosis in the index case. TB diagnosis was based on chest X-ray (CXR), positive sputum smear and culture.
Non-progressors	HHCs who remained healthy and did not develop TB during the follow-up period of 2 years. Negative for symptom screen, CXR, TST and IGRA.

### 2.3 Diagnostic tests for active TB

Sputum samples were collected from all the participants and subjected to GeneXpert MTB/RIF assay, followed by culture in Löwenstein-Jensen (LJ) medium and Mycobacterial Growth Indicator Tube (MGIT). Samples testing positive for *Mtb* using any of these methods were categorized as confirmed TB cases.

### 2.4 QuantiFERON-TB gold-in tube assay

Whole blood was collected and incubated in stimulated (TB antigen), positive control (mitogen), and unstimulated (Nil) tubes and processed as per the manufacturer's instructions (QIAGEN, Germany). After incubation, supernatants were harvested to measure the IFN-γ response (IU/mL). Individuals were considered QFT-positive or negative based on the analysis using the QFT-GIT analysis software (version 2.62). The remaining QuantiFERON supernatants were immediately stored at −80°C for further analysis.

### 2.5 RNA isolation

Total RNA was extracted from unstimulated and *Mtb* antigen-stimulated QuantiFERON supernatants using TRIzol reagent (Invitrogen, Waltham, MA, United States). RNA was quantified using the Qubit microRNA assay kit (Invitrogen) and the quality was assessed using the Agilent 2100 Bioanalyzer Pico chip (Agilent, Santa Clara, CA, United States).

### 2.6 NanoString miRNA assay

Three microlitres of extracted QuantiFERON supernatants containing 100 ng of total RNA was used for miRNA analysis using the NanoString Human v3A miRNA assay kit (NanoString Technologies, Seattle, WA, United States). miRNAs were then ligated to miRTag with ligase in ligation buffer supplied in the kit. The ligated product was diluted with 15 μl of nuclease-free water and denatured at 85°C for 5 min. Five microlitres of the mixture was hybridized overnight at 65°C with reporter and capture probes. Post hybridization, the samples were analyzed on a NanoString nCounter SPRINT machine.

### 2.7 miRNA expression analysis

miRNA profiles were analyzed in *Mtb* antigen-stimulated QuantiFERON supernatants collected at baseline (enrolment) for Non-progressors and at the timepoint closest to TB activation for Progressors using the nCounter Analysis System (NanoString Technologies) and the nCounter Human v3 miRNA panel having 799 unique clinically relevant miRNA barcodes for detecting endogenous miRNA, besides house-keeping genes, spike-in miRNAs, positive and negative controls to assess the overall efficiency of the run. The raw miRNA data in RCC (Reporter Code Count) format was analyzed using nSolver, v4.0 (NanoString Technologies). Normalization of the raw data was performed using the geometric mean of positive controls and top 100 highly expressed miRNAs (11–13). The use of highly expressed miRNAs for normalization provides robustness and consistency which helps to reduce the impact of outliers and low-abundance miRNAs that might introduce variability (14). Difference in miRNA expression between the groups was analyzed using the build ratio utility (foldchange). The threshold for significance for the differentially expressed miRNAs were foldchange ≥1.2 or ≤ −1.2, FDR adjusted *p*-value ≤ 0.05 and count ≥15.84, which is the average of the negative control.

### 2.8 Target prediction and functional enrichment analysis

Genes targeted by at least five of the significantly differentially regulated miRNAs were identified using the miRTarBase database in MIENTURNET tool (15). Functional pathway enrichment of the identified targets was performed using DAVID (16) with an EASE threshold of 0.05. A miRNA-target gene-pathway network displaying the top enriched pathways was built and visualized using Cytoscape (17). Further, the identified target genes were mapped in the TB pathogenesis pathway derived from Kyoto Encyclopedia of Genes and Genomes (KEGG) to understand their significance in TB pathogenesis (18–20).

### 2.9 Statistical analysis

Statistical analyses for this study were conducted using various R (21) packages including randomForest, caret, factoextra, combiroc, rstatix, readxl and ggplot2. Mann-Whitney test was used to compare between Progressors and Non-progressors, with a significance threshold set at *p* < 0.05.

The diagnostic potential of each miRNA was assessed using Receiver Operating Characteristic (ROC) analysis, with the area under the ROC curve (AUC) serving as a critical diagnostic index. ROC curves and AUC values were utilized to evaluate the specificity, diagnostic ability, and sensitivity of each miRNA, both individually and in combination.

The significance of dysregulated miRNAs was determined through Random Forest (RF) analysis. The dataset was split into training (75%) and test (25%) subsets for model training and evaluation. An iterative refinement process was applied to optimize classification accuracy. The initial RF model, which included all 30 dysregulated miRNAs, achieved an accuracy of 85%. In each iteration, miRNAs were sequentially removed based on their Gini Importance scores, reflecting their contribution to the model's performance. This process continued until the model attained a classification accuracy of 100%.

To evaluate the predictive performance of the trained model, Balanced Accuracy was used. Balanced Accuracy, which integrates both Sensitivity (true-positive rate) and Specificity (true-negative rate), is particularly useful for imbalanced datasets. It ranges from 0 to 1, with higher values indicating better overall model performance. Thus, Balanced Accuracy was chosen as the final performance evaluation metric. The accuracy of the selected miRNAs was further validated using an external validation cohort. Subsequently, Principal Component Analysis (PCA) was applied to the selected miRNAs to perform dimensionality reduction and identify underlying classification patterns.

## 3 Results

### 3.1 Characteristics of the study participants

**Figure 1:**
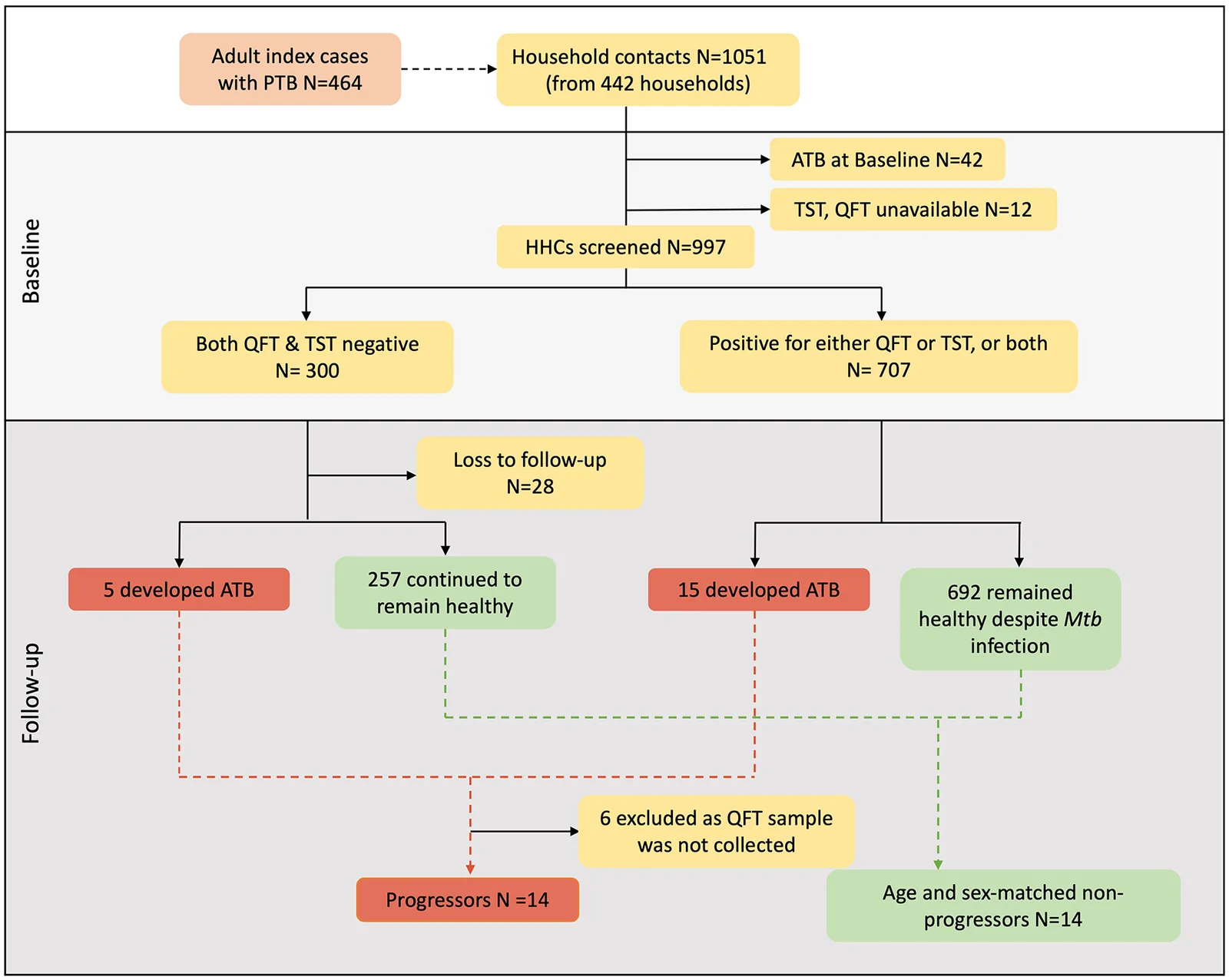
Participant selection from two sites of the C-TRIUMPh cohort study. A total of 1,051 adults and children were recruited in the C-TRIUMPh study. Participants were classified based on their baseline Mtb-infection status as positive for QFT (≥0.35 IU/ml) and/or positive for TST (induration diameter ≥5 mm) or negative for both. Among the enrolled participants who went on to develop TB during follow-up were identified as Progressors, and those who remained healthy were defined as Non-progressors. Progressors were matched to Non-progressors for age and gender.

The clinical characteristics of the participants who contributed samples for the miRNA analysis are provided in Table 2. Among the 1,051 HHCs from 442 households who were enrolled and followed-up for 2 years as part of the parent study, 20 individuals developed microbiologically confirmed TB within 2 years and were identified as Progressors; unfortunately, 6 of them did not have stored QuantiFERON supernatants and had to be excluded from the study. The duration between enrolment and diagnosis of active TB in the Progressors ranged from 3–21 months. An equal number of HHCs who did not develop active TB during the entire period of follow-up, matched for age and gender with the Progressors, were identified and selected as controls (Non-progressors) for the study. Figure 1 provides the flow chart depicting the selection of study participants.

**Table 2:** Clinical and demographic characteristics of the study population.

**Participant characteristics**	**Progressors (*n =* 14)**	**Non-progressors (*n =* 14)**
Age in years, median (IQR)^#^	32 (23–39)	35 (21–44)
**Sex**, ***n*** (%)^*^		
Male	5 (36)	4 (40)
Female	9 (64)	10 (60)

^#^*p*-value by Mann-Whitney test: 0.8121. ^*^p-value by Fisher's Exact test: 0.1283.

### 3.2 Differentially expressed miRNAs in Progressors, Non-progressors, and TB cases

**Figure 2:**
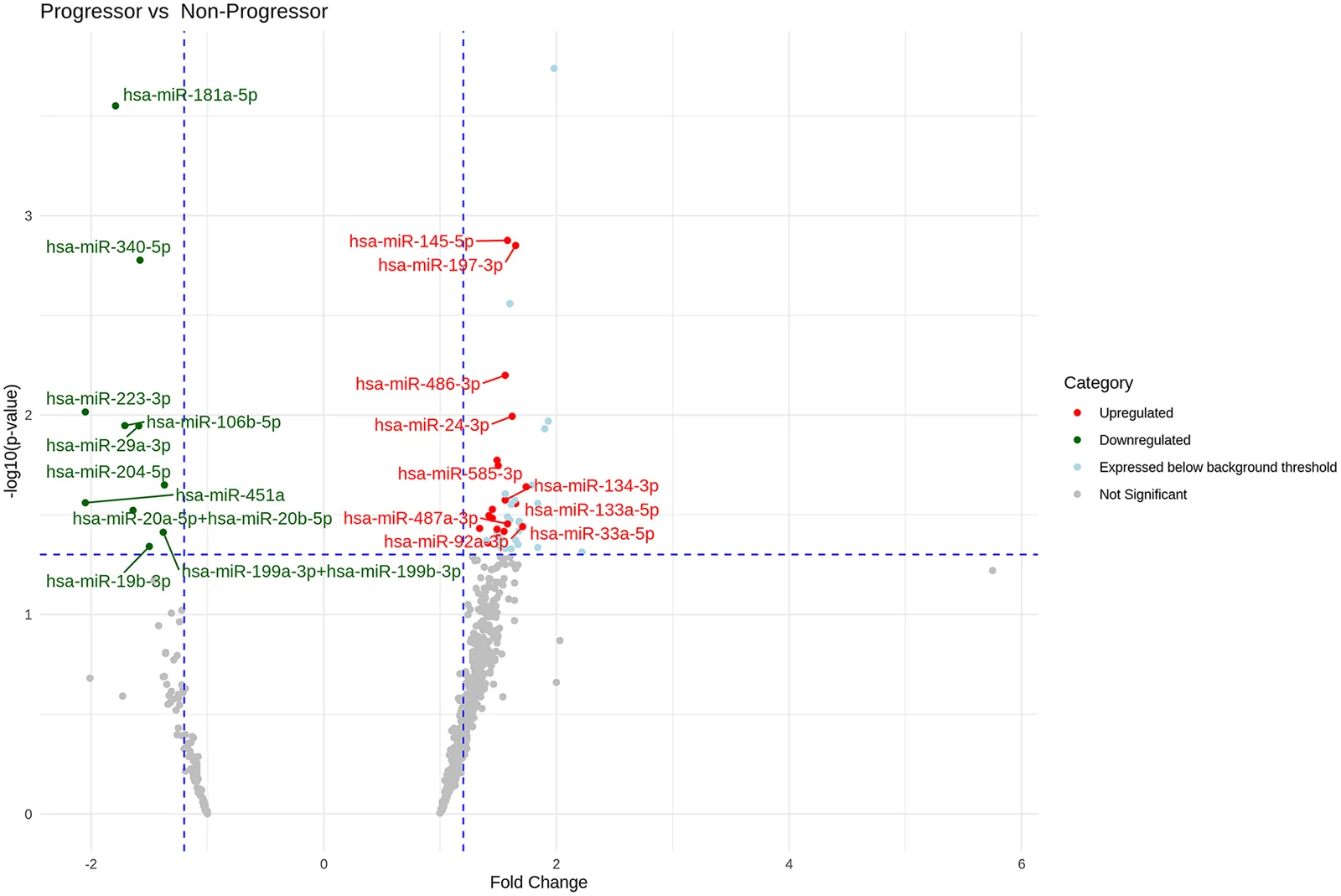
Volcano plot depicting upregulated (red dots) and downregulated (green dots) miRNAs between the Progressors and Non-progressors. Fold change of the 30 significantly dysregulated miRNAs in the study groups. The threshold considered for significance was fold change ≥1.2 or ≤ −1.2 with FDR adjusted *p*-value ≤ 0.05. The top 10 upregulated and downregulated miRNAs are labeled. Blue dots represent miRNAs expressed below the average count of the negative control.

Of the 799 miRNAs analyzed using NanoString, 30 miRNAs showed significantly differential expression between the Progressors and Non-progressors. Of these, 20 miRNAs were up-regulated and 10 were down-regulated. Figure 2 represents the fold change of the significantly altered miRNAs between the two groups. Among the upregulated miRNAs, hsa-miR-585-3p and hsa-miR-92a-3p showed the maximum fold change of 1.74 and 1.71 respectively. Among the downregulated miRNAs, hsa-miR-223-3p and hsa-miR-451a exhibited the maximum fold change of −2.05 and −2.04 folds respectively.

### 3.3 Identification of miRNA targets and altered biological pathways

**Figure 3:**
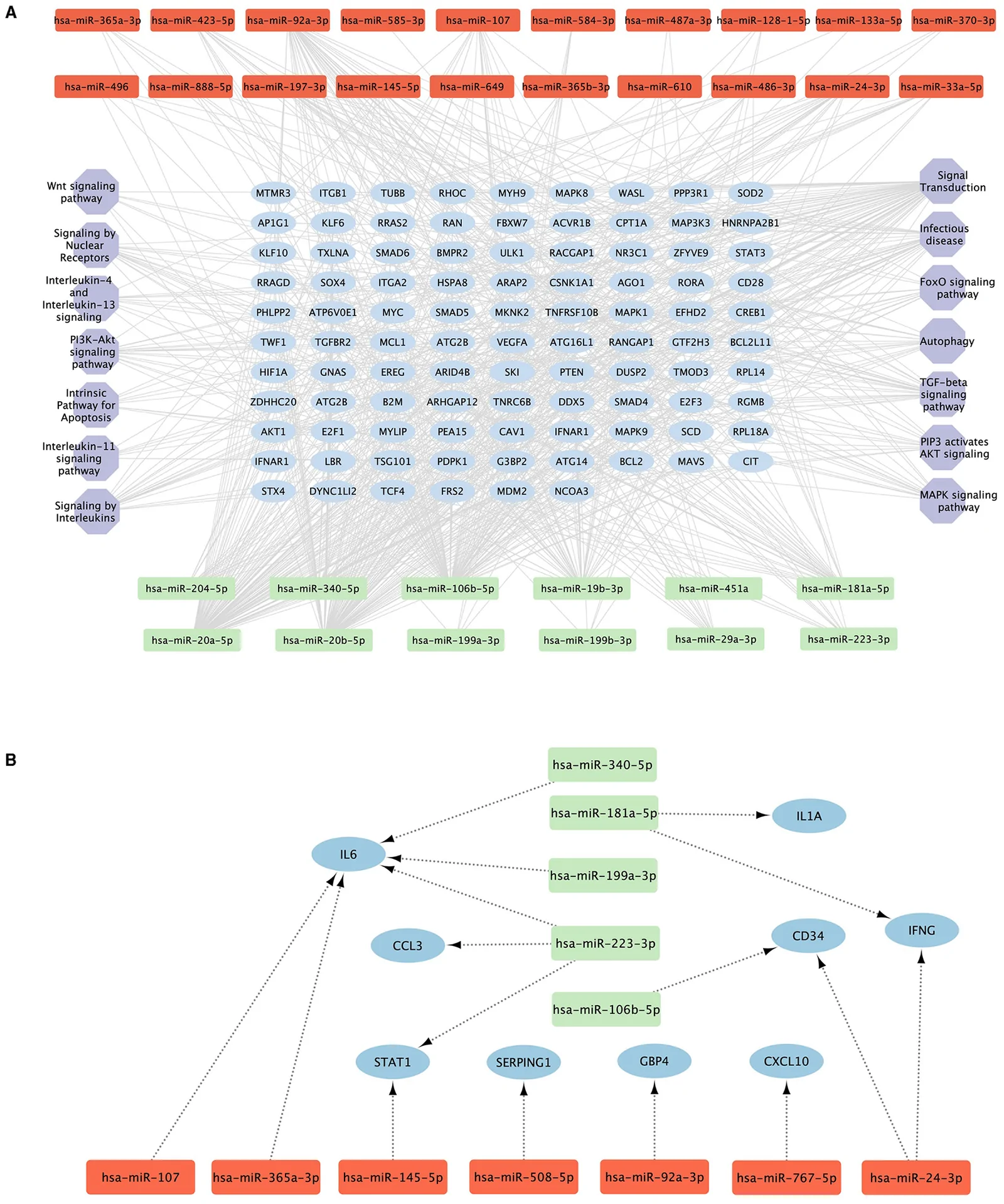
Pathways targeted by the significantly altered miRNAs. **(A)** The top 5 hub genes (blue ellipses) targeted by the 30 differentially expressed upregulated (red) and downregulated (green) miRNAs with a minimum interaction of five and their corresponding pathways (purple hexagons). **(B)** Previously reported TB predictive genes (blue ellipses) targeted by the significantly upregulated (red) and downregulated (green) miRNAs.

We explored the role of the 30 miRNAs that were significantly different between the Progressors and Non-progressors in the pathogenesis/progression of TB. For this, we set a minimum interaction threshold of five in the MIENTURNET tool and identified 234 mRNA targets from the miRTarBase database. The identified targets were uploaded on DAVID database with an EASE threshold of 0.05 for pathway analysis. The analysis revealed that the miRNA targets were significantly enriched in autophagy, TGF-beta signaling, FOXO signaling, apoptosis and Interleukin signaling pathways (Figure 3A). Supplementary Table 1 provides a list of all the pathways identified using DAVID database. Very interestingly, 12 of these miRNAs were found to target previously reported TB susceptibility-associated genes. For example, five miRNAs *viz*. hsa-miR-340-5p, hsa-miR-199a-3p, hsa-miR-223-3p, hsa-365a-3p, and hsa-miR-107 were found to target the proinflammatory cytokine gene IL6 (Figure 3B).

### 3.4 Diagnostic performance of the significantly dysregulated miRNAs

**Figure 4:**
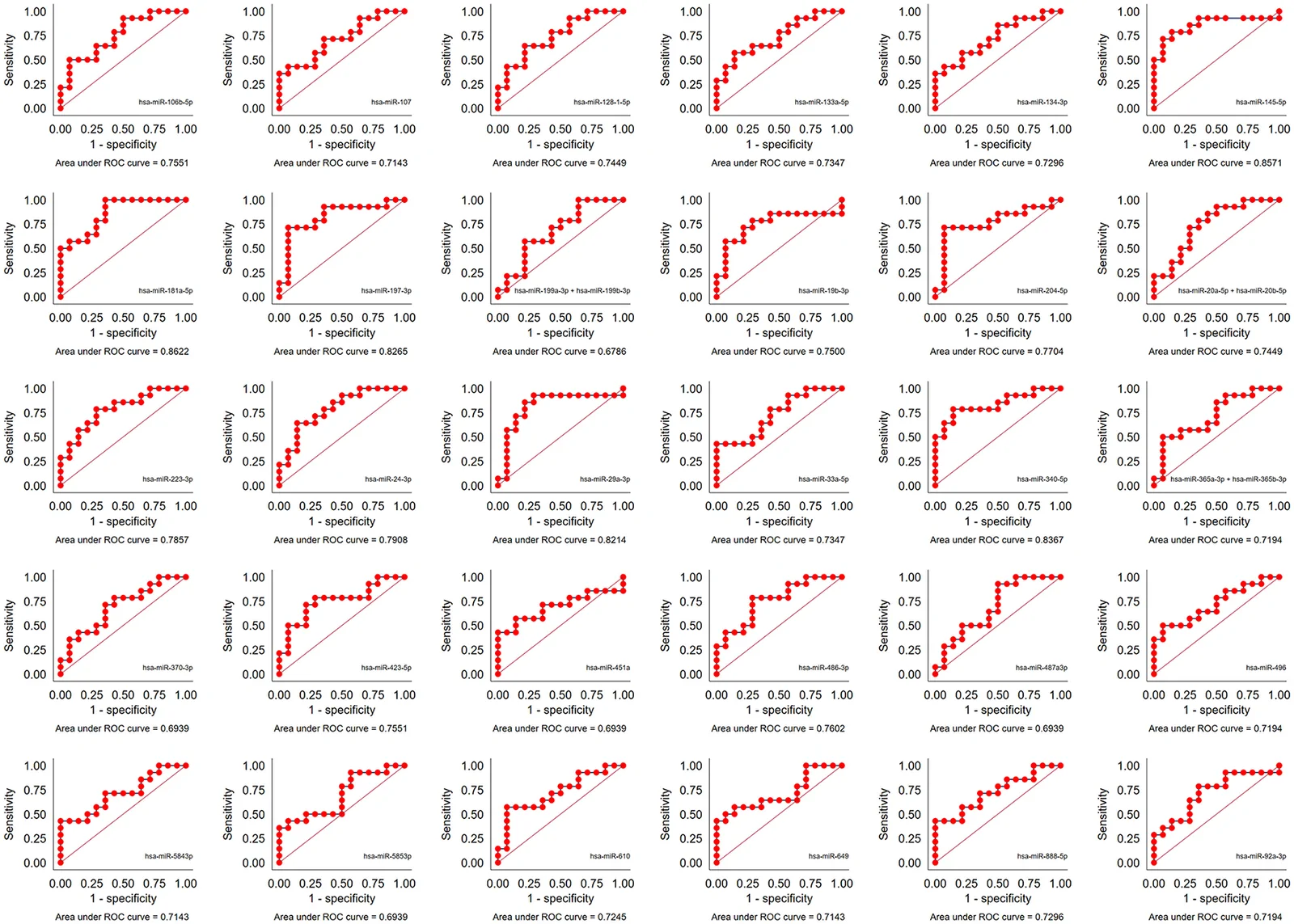
Performance of the 30 significantly dysregulated miRNAs in predicting TB progression. Area under the receiver operator curve (AUC) for all the 30 miRNAs.

ROC analysis was performed to determine the diagnostic accuracy of the 30 significantly different miRNAs between the Progressors and Non-progressors. hsa-miR-181a-5p demonstrated maximum accuracy with an area under the curve (AUC) of 0.86, sensitivity of 73.68% (95%CI 54.43–90.85) and specificity of 100% (95% CI 66.37–100.00). Additionally, hsa-miR-204-5p, hsa-miR-197-3p, hsa-miR-340-5p, hsa-miR-145-5p, and hsa-miR-29a-3p demonstrated >75% sensitivity and specificity (Figure 4, Table 3).

**Table 3:** Diagnostic performance of the significantly dysregulated miRNAs.

**miRNA**	**Sensitivity % (95% CI)**	**Specificity % (95% CI)**	**PPV % (95% CI)**	**NPV % (95% CI)**	**AUC % (95% CI)**
hsa-miR-181a-5p	73.68 (54.43–90.85)	100.00 (66.37–100.00)	100.00 (76.84–100.00)	64.29 (41.90–87.24)	86.84 (76.67–97.01)
hsa-miR-204-5p	90.91 (71.51–99.77)	76.47 (56.67–93.19)	71.43 (49.20–91.61)	92.86 (76.84–99.82)	83.69 (70.00–97.38)
hsa-miR-197-3p	90.91 (71.51–99.77)	76.47 (56.67–93.19)	71.43 (49.20–91.61)	92.86 (76.84–99.82)	83.69 (70.00–97.38)
hsa-miR-340-5p	84.62 (63.97–98.08)	80.00 (59.54–95.67)	78.57 (57.19–95.34)	85.71 (66.13–98.22)	82.31 (67.68–96.93)
hsa-miR-33a-5p	100.00 (54.07–100.00)	63.64 (45.13–82.80)	42.86 (23.04–71.14)	100.00 (76.84–100.00)	81.82 (71.53–92.11)
hsa-miR-584-3p	100.00 (54.07–100.00)	63.64 (45.13–82.80)	42.86 (23.04–71.14)	100.00 (76.84–100.00)	81.82 (71.53–92.11)
hsa-miR-888-5p	100.00 (54.07–100.00)	63.64 (45.13–82.80)	42.86 (23.04–71.14)	100.00 (76.84–100.00)	81.82 (71.53–92.11)
hsa-miR-145-5p	84.62 (63.97–98.08)	76.47 (56.67–93.19)	73.33 (51.91–92.21)	86.67 (68.05–98.34)	80.54 (65.98–95.11)
hsa-miR-29a-3p	76.47 (56.57–93.19)	84.62 (63.97–98.08)	86.67 (68.05–98.34)	73.33 (51.91–92.21)	80.54 (65.98–95.11)
hsa-miR-610	88.89 (66.37–99.72)	68.42 (48.80–87.42)	57.14 (35.14–82.34)	92.86 (76.84–99.82)	78.65 (63.36–93.95)
hsa-miR-487a-3p	65.00 (45.72–84.61)	87.50 (63.06–99.68)	92.86 (76.84–99.82)	50.00 (28.86–76.96)	76.25 (59.97–92.53)
hsa-miR-451a	87.50 (63.06–99.68)	65.00 (45.72–84.61)	50.00 (28.86–76.96)	92.86 (76.84–99.82)	76.25 (59.97–92.53)
hsa-miR-365a-3p + hsa-miR-365b-3p	87.50 (63.06–99.68)	65.00 (45.72–84.61)	50.00 (28.86–76.96)	92.86 (76.84–99.82)	76.25 (59.97–92.53)
hsa-mir-496	87.50 (63.06–99.68)	65.00 (45.72–84.61)	50.00 (28.86–76.96)	92.86 (76.84–99.82)	76.25 (59.97–92.53)
hsa-miR-649	87.50 (63.06–99.68)	65.00 (45.72–84.61)	50.00 (28.86–76.96)	92.86 (76.84–99.82)	76.25 (59.97–92.53)
hsa-miR-24-3p	81.82 (58.72–97.72)	70.59 (50.10–89.69)	64.29 (41.90–87.24)	85.71 (66.13–98.22)	76.20 (59.85–92.56)
hsa-miR-19b-3p	76.92 (54.55–94.96)	73.33 (51.91–92.21)	71.43 (49.20–91.61)	78.57 (57.19–95.34)	75.13 (58.51–91.75)
hsa-miR-223-3p	73.33 (51.91–92.21)	76.92 (54.55–94.96)	78.57 (57.19–95.34)	71.43 (49.20–91.61)	75.13 (58.51–91.75)
hsa-miR-486-3p	73.33 (51.91–92.21)	76.92 (54.55–94.96)	78.57 (57.19–95.34)	71.43 (49.20–91.61)	75.13 (58.51–91.75)
hsa-miR-423-5p	73.33 (51.91–92.21)	73.33 (51.91–92.21)	73.33 (51.91–92.21)	73.33 (51.91–92.21)	73.33 (56.95–89.71)
hsa-miR-133a-5p	80.00 (55.50–97.48)	66.67 (46.52–86.66)	57.14 (35.14–82.34)	85.71 (66.13–98.22)	73.33 (56.12–90.55)
hsa-miR-92a-3p	68.75 (47.62–88.98)	75.00 (51.59–94.51)	78.57 (57.19–95.34)	64.29 (41.90–87.24)	71.88 (54.52–89.23)
hsa-miR-1281-5p	75.00 (51.59–94.51)	68.75 (47.62–88.98)	64.29 (41.90–87.24)	78.57 (57.19–95.34)	71.88 (54.52–89.23)
hsa-miR-106b-5p	71.43 (49.20–91.61)	71.43 (49.20–91.61)	71.43 (49.20–91.61)	71.43 (49.20–91.61)	71.43 (54.06–88.79)
hsa-miR-20a-5p + hsa-miR-20b-5p	66.67 (46.52–86.66)	75.00 (51.59–94.51)	80.00 (59.54–95.67)	60.00 (38.38–83.66)	70.83 (53.83–87.84)
hsa-miR-107	77.78 (51.75–97.19)	63.16 (43.45–83.71)	50.00 (28.86–76.96)	85.71 (66.13–98.22)	70.47 (52.26–88.68)
hsa-miR-134-3p	72.73 (48.22–93.98)	64.71 (44.04–85.79)	57.14 (35.14–82.34)	78.57 (57.19–95.34)	68.72 (50.62–86.82)
hsa-miR-199a-3p + hsa-miR-199b-3p	64.71 (44.04–85.79)	72.73 (48.22–93.98)	78.57 (57.19–95.34)	57.14 (35.14–82.34)	68.72 (50.62–86.82)
hsa-miR-585-3p	72.73 (48.22–93.98)	64.71 (44.04–85.79)	57.14 (35.14–82.34)	78.57 (57.19–95.34)	68.72 (50.62–86.82)
hsa-miR-370-3p	64.71 (44.04–85.79)	69.23 (46.19–90.91)	73.33 (51.91–92.21)	60.00 (38.38–83.66)	66.97 (49.43–84.51)

**Figure 5:**
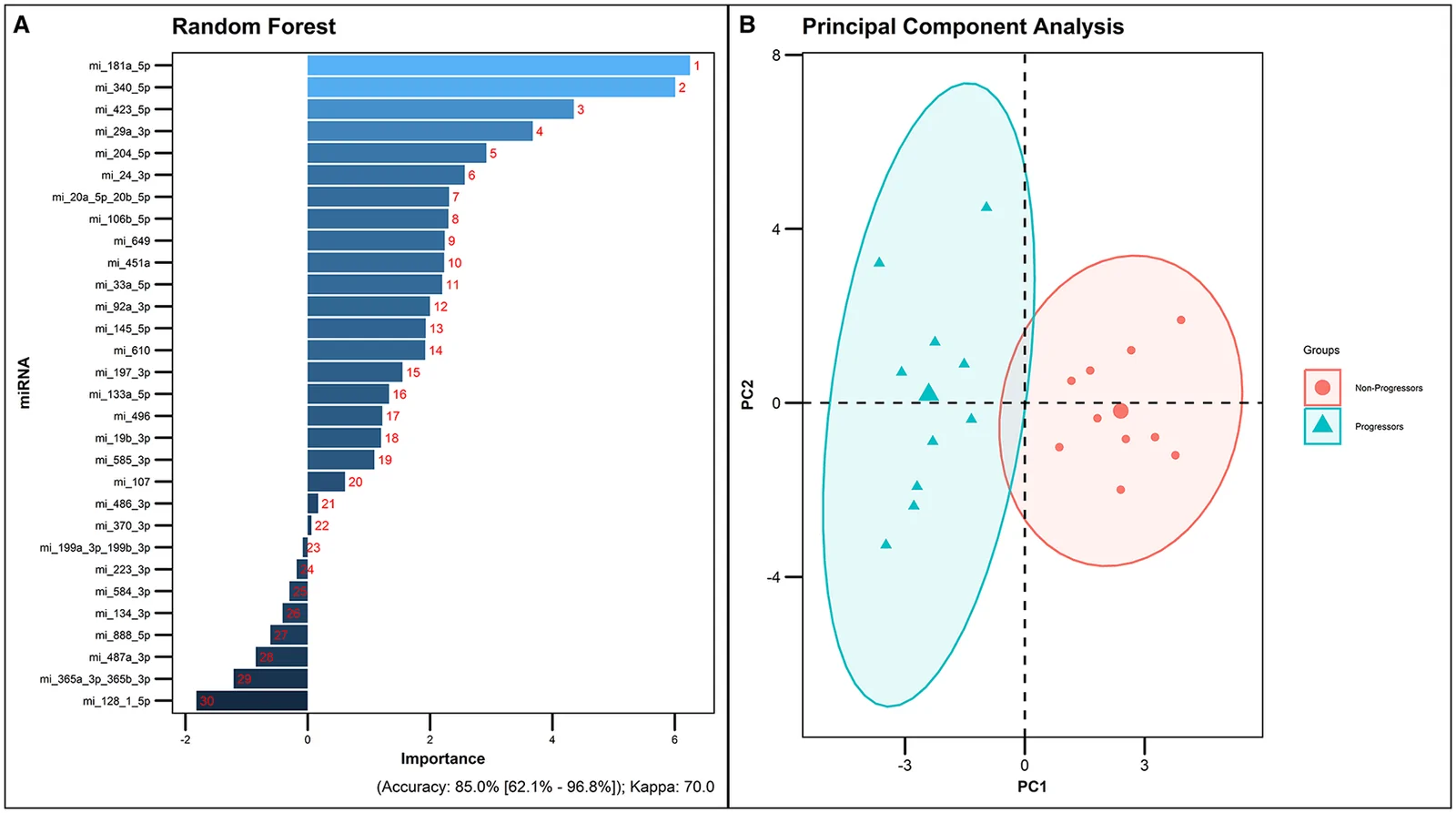
Random forest analysis of the 30 significantly dysregulated miRNAs. **(A)** miRNAs ranked by the order of their predictive importance. **(B)** PCA plot of the 30 differentially expressed miRNAs in the Progressors and Non-progressors (FDR-adjusted *p* ≤ 0.05).

Random forest analysis was performed using R package randomForest with default settings. The data set was split into 75/25 for training and testing. This analysis ranked the 30 miRNAs based on their importance. The results of the RF analysis was found to be in accordance with the ROC analysis where the top 10 significantly different miRNAs exhibited an AUC >0.7. The overall predictive accuracy of the 30 miRNAs according to RF analysis was 85% (Figure 5).

**Figure 6:**
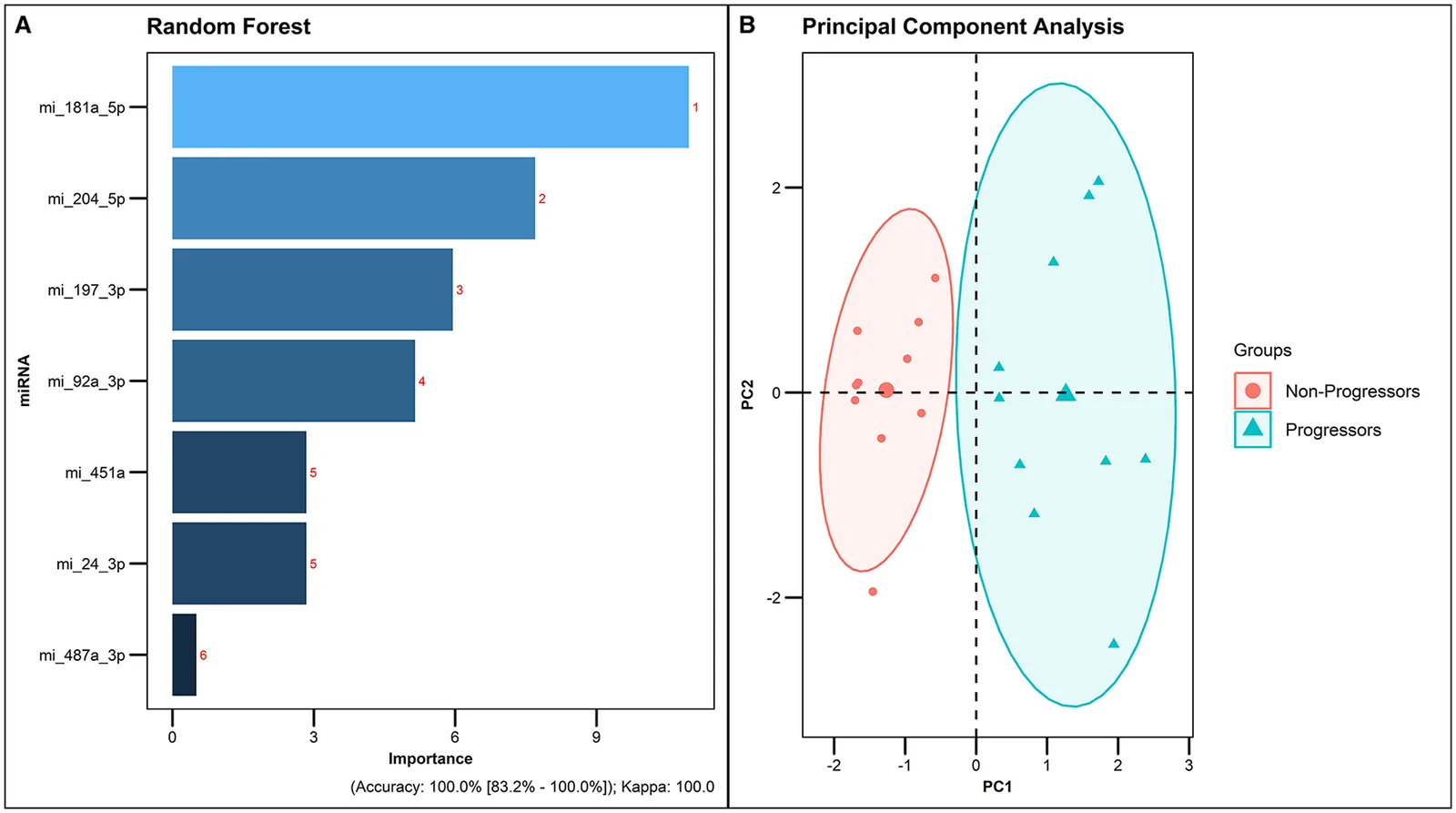
Best predictive model generated by Random forest analysis. **(A)** Predictive accuracy of the best model generated by Random forest analysis. **(B)** PCA plot of the 7 differentially expressed miRNAs in Progressors and Non-progressors (FDR-adjusted *p* ≤ 0.05).

In order to enhance the predictive accuracy of the shortlisted miRNAs and to identify the optimal predictive miRNA model, dimensionality reduction was performed using Principal Component Analysis using default settings in the R package. Notably, hsa-miR-181a-5p, hsa-miR-204-5p, hsa-miR-197-3p, hsa-miR-92a-3p, hsa-miR-451a, hsa-miR-24-3p, and hsa-miR-487a-3p exhibited a remarkable 100% predictive accuracy in the analysis. The amalgamation of these seven miRNAs showcased a perfect 100% sensitivity, specificity and positive predictive value, underscoring their potential as a robust predictive model (Figure 6).

### 3.5 External validation of the 7-miRNA signature

**Figure 7:**
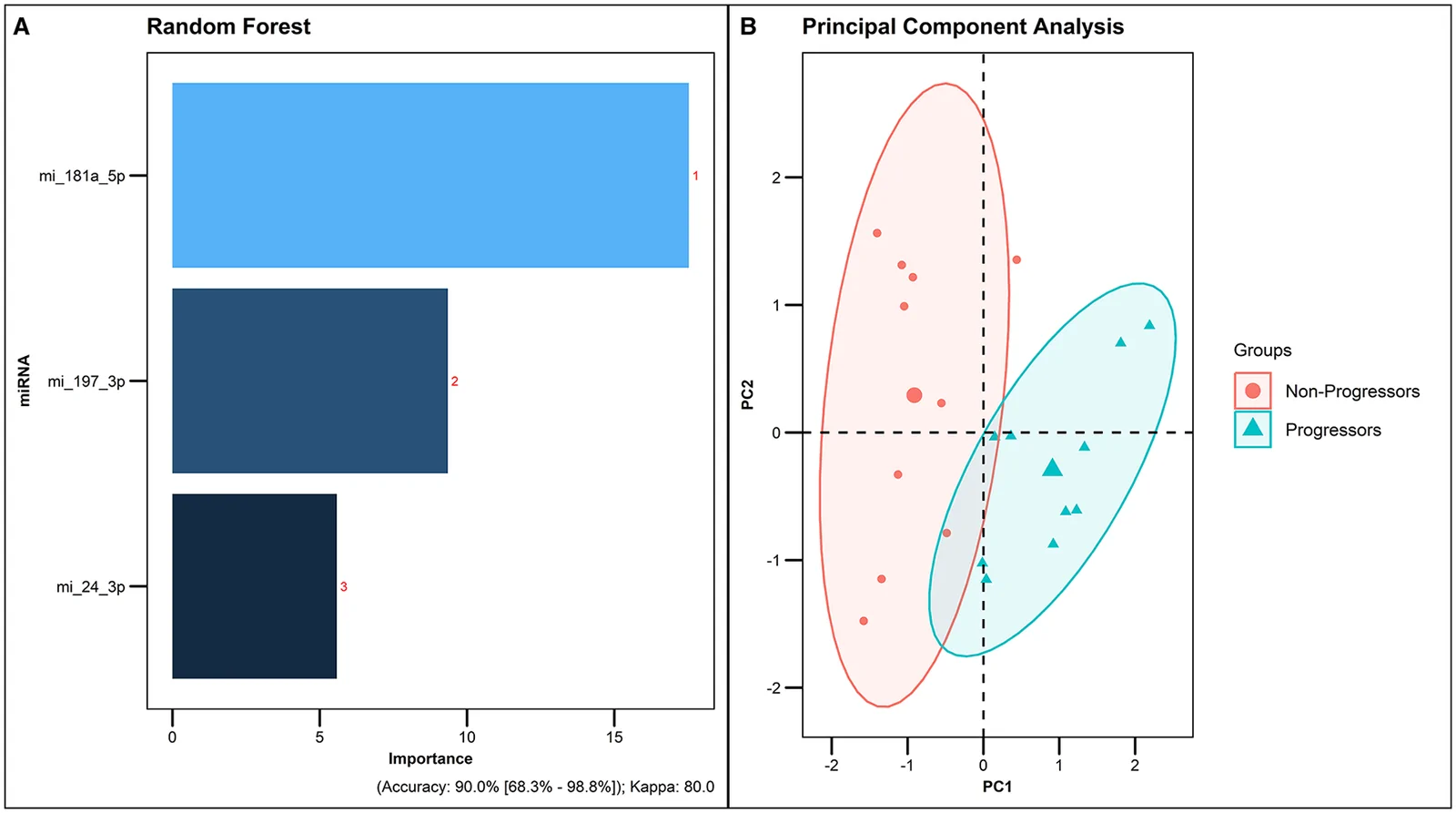
Predictive accuracy of the combination of 3 common miRNAs from the external dataset. Duffy et al. (22) as a testing cohort and our study dataset for model training. **(A)** Predictive accuracy of the model generated by Random forest analysis. **(B)** PCA plot of the 3 common dysregulated miRNAs in Progressors and Non-progressors.

To further validate the results obtained from the Random Forest Analysis, we employed an external dataset from Duffy et al. (22) as a test cohort, while utilizing our study dataset for model training. In this validation process, we concentrated on three microRNAs common to both datasets: hsa-miR-181a-5p, hsa-miR-197-3p, and hsa-miR-24-3p. The analysis of these miRNAs, when integrated into the predictive model, demonstrated a high accuracy rate of 90% (Figure 7). This high level of accuracy not only confirms the robustness of our predictive model but also highlights the potential of these miRNAs as key biomarkers for tuberculosis pathogenesis and prognosis.

### 3.6 Implication of the seven predictive miRNAs in TB pathogenesis

**Figure 8:**
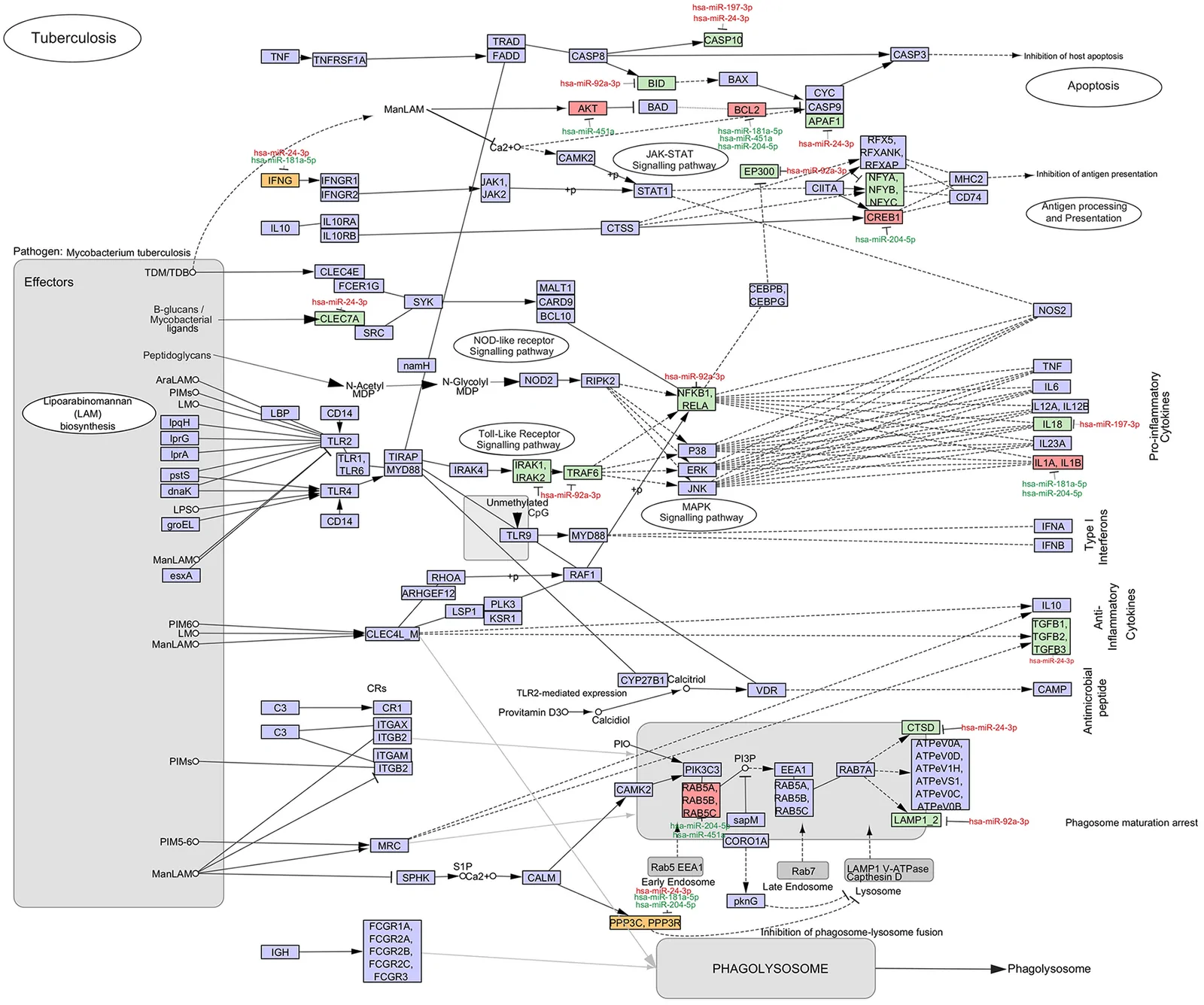
Significance of the dysregulated miRNAs in TB pathogenesis. The mRNA targets of the identified seven miRNAs were mapped in the KEGG pathway for tuberculosis. The upregulated (red) and downregulated (green) miRNAs and mRNAs in the QuantiFERON supernatants of Progressors vs. Non-progressors are mapped. The mRNA in yellow boxes are targeted by both an upregulated and downregulated miRNA. mRNAs in purple boxes are not targeted by any of the seven identified miRNAs.

To fully understand the significance of the identified 7-miRNA signature in the pathogenesis of tuberculosis, their respective targets were analyzed in the KEGG pathway for Tuberculosis. Very interestingly, except for hsa-miR-487a-3p, all the other miRNAs were found to target multiple genes implicated in different pathways pertaining to the pathogenesis of tuberculosis (Figure 8).

## 4 Discussion

Several recent studies have established the fact that miRNAs are among the critical modulators of the immune response to *Mtb* (23–25). Altered miRNA expression profiles that differentiate active TB from latent TB have been reported in several studies, suggesting that miRNAs have the potential to serve as reliable biomarkers for TB (24). Based on the order of predictive importance, hsa-miR-181a-5p, hsa-miR-204-5p, hsa-miR-197-3p, hsa-miR-92a-3p, hsa-miR-451a, hsa-miR-24-3p, and hsa-miR-487a-3p were identified as the most promising biomarkers exhibiting superlative sensitivity and specificity for predicting risk of progression to infectious TB disease.

Since miRNAs circumvent many of the disadvantages of the currently available prognostic tests for TB, we attempted to explore the regulatory role of miRNAs in TB disease progression and identified highly promising TB-specific miRNAs with excellent predictive ability. Mapping the mRNA targets of these miRNAs in the TB pathogenesis pathway provided an insight into the possible underlying regulatory role of these miRNAs in disease progression. Apoptosis is an important host defense mechanism in the pathogenesis of several diseases including tuberculosis. Several miRNAs identified in our analysis were found to target key genes in the apoptotic pathway. Up-regulation of hsa-miR-197-3p and hsa-miR-24-3p causes down-regulation of CASP10, an initiator of the caspase cascade leading to inhibition of apoptosis. Increased expression of hsa-miR-92a-3p leads to BID activation, which leads to inhibit the Bax-Bak mediated intrinsic apoptotic pathway (26). Up-regulation of hsa-miR-24-3p causes a reduction in the expression of APAF1, a subunit of a multiprotein complex called casposome often referred to as the “death-inducing signaling complex” (DISC) (27). Decreased expression of hsa-miR-451a promotes the phosphorylation of AKT which leads to the suppression of BAD, a pro-apoptotic factor (28). A major gene targeted by three of the seven significantly altered miRNAs is BCL2, which is an anti-apoptotic regulatory element. Up-regulation of hsa-miR-181a-5p, hsa-miR-451a, and hsa-miR-204-5p increases BCL2 expression. A recent study demonstrated that MCL-1 and BCL-2 inhibitors induce apoptosis to control *Mtb* growth in macrophages and suggests that MCL-1 and BCL-2 may be promising targets for host-directed therapy for tuberculosis (29). Put together, our findings reveal a key role for the identified predictive miRNAs in inhibiting apoptosis thereby contributing to progression of *Mtb* infection to infectious TB disease.

The other two crucial pathways targeted by the identified miRNAs include the Toll-like receptor pathway and MAPK pathway. MiRNA hsa-miR-92a-3p dampens the expression of five important genes in these pathways. Nuclear factor kappa B (NF-κB) is known to play a unique role in mediating the proinflammatory response against *Mtb* by promoting the activation of macrophages and secretion of cytokines like TNF-α, IL-1, IL-6, and IL-12, that play a crucial role in keeping the granuloma intact and the bacteria contained. Targeting of the IL-1 receptor-associated kinase 1 and 2 (IRAK1 and IRAK2) genes by hsa-miR-92a-3p inhibits the phosphorylation of tumor necrosis factor (TNF) receptor-associated factor (TRAF) and interferes with the NF-κB signaling pathway (30), which is important for pro-inflammatory responses and containment of TB disease, thereby contributing to TB reactivation and disease progression.

Increased expression of has-miR-92a-3p as seen in Progressors may lead to increased binding to the Nuclear transcription Factor Y (NFY) complex, consisting of the NFYa, NFYb, and NFYc subunits, and thereby impede MHC class I antigen presentation (31). On the other hand, downregulation of hsa-miR-204-5p in Progressors leads to the increased expression of cyclic AMP Response Element Binding Protein 1 (CREB1) which has been shown to play a key role in immune evasion of *Mtb* in human macrophages (32).

Three of the identified miRNAs target key cytokine genes. Hsa-miR-197-3p targets IL18 and decreases its production, while hsa-miR-181a-5p and hsa-miR-204-5p up-regulate IL1A and IL1B production. The anti-inflammatory cytokine, TGF-β is down-regulated by the regulatory effect of hsa-miR-24-3p which targets TGFB1, TGFB2, and TGFB3. IFNG is targeted by both an up-regulated miRNA, hsa-miR-24-3p, and a down-regulated miRNA, hsa-miR-181a-5p. This might indicate a tilt in the fine balance in IFN-γ production and downstream responses during disease progression. A C-type lectin receptor called Dectin-1 (CLEC7A), which recognizes *Mtb* glucan is negatively regulated by hsa-miR-24-3p which impairs the production of inflammatory cytokines that are crucial for protection against *Mtb* (33, 34).

A number of miRNAs involved in the phagosome maturation pathway are dysregulated in Progressors. Hsa-miR-204-5p and hsa-miR-451a target RAB5A, RAB5B, RAB5C, and abrogate phagosome maturation (35). Hsa-miR-24-3p down-regulates the lysosome marker Cathepsin D encoded by the CTSD gene and hsa-miR-92a-3p down-regulates the lysosome-associated membrane proteins encoded by LAMP1 and LAMP2 (36). Hsa-miR-24-3p, hsa-miR-181a-5p, and hsa-miR-204-5p are known to regulate PPP3C (Calcineurin A) and PPP3R (Calcineurin B) thereby preventing phagosome-lysosome fusion (37). Though there is no *in vitro* data to support the role of hsa-miR-24-3p in the pathogenesis of tuberculosis, a recent study that elucidated the interactive network of hsa-miR-24–3p-NEAT1 [a long non-coding RNA (LncRNA)]-Adrenomedullin (ADM)-CEBPB (a transcription factor), found it to be a critical pathway in the regulation of TB pathogenesis (38).

As much as the host tries to contain the bacterium, it is intriguing to note that *Mtb* can re-pattern the entire host immune system in order to set up a favorable niche for its survival and growth. The shift in macrophage polarization from M1 to the immune compromised M2 phenotype which is characterized by the production of Th2, Treg, and Th17 cytokines, gives *Mtb* an upper hand to persist and reactivate (39, 40). It is evident from our findings that the significantly dysregulated miRNAs in Progressors mainly target pathways that produce Th2, Treg, and Th17 cytokines which play a crucial role in TB reactivation.

Interestingly earlier studies have also described the utility of three of the miRNAs shortlisted in our study as biomarkers for TB. Significantly increased serum levels of hsa-miR-197-3p has previously been reported in TB patients as compared to healthy controls with an AUC of 0.84 (41). Hsa-miR-451a has been suggested as a promising biomarker in combination with body mass index (BMI) and prior history of TB, with a sensitivity of 80.82% and a specificity of 79.22% for identifying individuals at risk of developing TB in a Chinese population (42). Hsa-miR-204-5p has been identified as a putative prognostic marker in non-small cell lung cancer (43, 44).

Duffy et al. published the first report on a circulating miRNA signature for TB progression based on published literature and showed that a 3 miRNA signature comprising of hsa-miR-148b-3p+hsa-miR-21-5p+hsa-miR-484 could predict TB progression within 6 months prior to development of disease with an AUC of 0.74 and up to 24 months before disease break-out with an AUC of 0.66 (22).

We for the first time analyzed QuantiFERON supernatants of Progressors and identified a set of seven highly promising TB-specific circulating miRNA biomarkers (hsa-miR-181a-5p, hsa-miR-24-3p, hsa-miR-92a-3p, hsa-miR-204-5p, hsa-miR-451a, hsa-miR-197-3p, and hsa-miR-487a-3p) capable of predicting risk of progression to active TB with excellent predictive accuracy reaching the Target Product Profile (TPP) recommended by WHO for tests for predicting progression from TB infection to disease (4). There are however a few limitations in our study. To support the validity of our findings, we examined the correlation between our PC1 scores with clinical and demographic variables, including gender, age, body mass index, IGRA results, Mantoux test results, BCG vaccination status, current and past smoking status, alcohol consumption, diabetes, and HIV status. But we could not observe any significant association with any of these variables probably because of the small sample size. Hence, this miRNA signature needs validation in multiple independent cohorts to assess their clinical utility and predictive performance. We could not validate this signature in an independent cohort as part of this study due to non-availability of a similar well-characterized longitudinal cohort from our population.

Once validated, the miRNA biosignature can be translated into an easy to use point-of-care test using advanced technologies like microfluidics, nanotechnology, and biosensors which are driving the development of more sophisticated Point-Of-Care tests (45). Integrating this miRNA signature as a predictive test in clinical settings can aid in the accurate identification of high-risk individuals and orienting them for treatment.

## 5 Conclusion

In summary, our study presents the first investigation of miRNAs from QuantiFERON supernatants as potential biomarkers for TB progression. Herein, we identified 30 differentially regulated circulating miRNAs in TB-antigen stimulated QuantiFERON supernatants of Progressors using the nanostring platform and identified a highly promising TB-specific 7 miRNA signature (hsa-miR-181a-5p + hsa-miR-24-3p + hsa-miR-92a-3p + hsa-miR-204-5p + hsa-miR-451a + hsa-miR-197-3p + hsa-miR-487a-3p) that can predict progression from tuberculosis infection to active TB disease. The identified miRNA signature warrants further validation in diverse multi-country cohorts for their performance in identifying individuals with the highest risk of progression to active TB, so that it can be developed into a rapid screening test that would be useful for planning targeted interventions for TB control.

## Data Availability

The datasets presented in this study can be found in online repositories. The names of the repository/repositories and accession number(s) can be found in the article/Supplementary material.
