# Supplementary material for: Discovery of a blood-based miRNA signature that can predict onset of active tuberculosis among household contacts of TB patients
Source: Front Tuberc. 2024 Oct 29;2:1415346. doi: 10.3389/ftubr.2024.1415346 (PMC13495009; doi:10.3389/ftubr.2024.1415346)
Supplement: Supplementary file 1 [file Table_1.DOCX]

**Supplementary Table 1**

**Pathways identified by DAVID database for the 234 mRNA targets of the 30 significantly dysregulated miRNAs**

| **Category** | **Term** | **Count** | **%** | **PValue** |
| --- | --- | --- | --- | --- |
| KEGG_PATHWAY | hsa05166:Human T-cell leukemia virus 1 infection | 30 | 4.69 | 2.41E-09 |
| WIKIPATHWAYS | WP437:EGF EGFR signaling pathway | 26 | 4.07 | 3.24E-09 |
| REACTOME_PATHWAY | R-HSA-162582~Signal Transduction | 153 | 23.94 | 6.40E-09 |
| KEGG_PATHWAY | hsa04140:Autophagy - animal | 25 | 3.91 | 7.22E-09 |
| KEGG_PATHWAY | hsa04218:Cellular senescence | 23 | 3.60 | 5.53E-08 |
| REACTOME_PATHWAY | R-HSA-6785807~Interleukin-4 and Interleukin-13 signaling | 19 | 2.97 | 1.47E-07 |
| WIKIPATHWAYS | WP3888:VEGFA VEGFR2 signaling | 42 | 6.57 | 1.65E-07 |
| WIKIPATHWAYS | WP366:TGF beta signaling pathway | 21 | 3.29 | 2.37E-07 |
| KEGG_PATHWAY | hsa05161:Hepatitis B | 22 | 3.44 | 4.78E-07 |
| WIKIPATHWAYS | WP5353:Macrophage stimulating protein MSP signaling network map | 16 | 2.50 | 6.70E-07 |
| REACTOME_PATHWAY | R-HSA-168256~Immune System | 120 | 18.78 | 1.15E-06 |
| WIKIPATHWAYS | WP3646:Hepatitis C and hepatocellular carcinoma | 13 | 2.03 | 1.55E-06 |
| WIKIPATHWAYS | WP4263:Pancreatic adenocarcinoma pathway | 16 | 2.50 | 1.99E-06 |
| KEGG_PATHWAY | hsa05163:Human cytomegalovirus infection | 25 | 3.91 | 2.66E-06 |
| KEGG_PATHWAY | hsa05212:Pancreatic cancer | 14 | 2.19 | 3.50E-06 |
| REACTOME_PATHWAY | R-HSA-1643685~Disease | 107 | 16.74 | 4.30E-06 |
| REACTOME_PATHWAY | R-HSA-1632852~Macroautophagy | 19 | 2.97 | 4.74E-06 |
| KEGG_PATHWAY | hsa05200:Pathways in cancer | 42 | 6.57 | 4.84E-06 |
| WIKIPATHWAYS | WP4806:EGFR tyrosine kinase inhibitor resistance | 15 | 2.35 | 5.02E-06 |
| REACTOME_PATHWAY | R-HSA-9612973~Autophagy | 20 | 3.13 | 5.53E-06 |
| WIKIPATHWAYS | WP5094:Orexin receptor pathway | 15 | 2.35 | 8.81E-06 |
| KEGG_PATHWAY | hsa05167:Kaposi sarcoma-associated herpesvirus infection | 22 | 3.44 | 9.04E-06 |
| KEGG_PATHWAY | hsa05219:Bladder cancer | 10 | 1.56 | 1.41E-05 |
| KEGG_PATHWAY | hsa04933:AGE-RAGE signaling pathway in diabetic complications | 15 | 2.35 | 1.66E-05 |
| KEGG_PATHWAY | hsa05169:Epstein-Barr virus infection | 22 | 3.44 | 1.69E-05 |
| REACTOME_PATHWAY | R-HSA-8953897~Cellular responses to stimuli | 57 | 8.92 | 1.82E-05 |
| REACTOME_PATHWAY | R-HSA-5663205~Infectious disease | 67 | 10.49 | 1.93E-05 |
| KEGG_PATHWAY | hsa05205:Proteoglycans in cancer | 22 | 3.44 | 2.12E-05 |
| REACTOME_PATHWAY | R-HSA-2262752~Cellular responses to stress | 56 | 8.76 | 2.22E-05 |
| KEGG_PATHWAY | hsa05206:MicroRNAs in cancer | 28 | 4.38 | 2.84E-05 |
| WIKIPATHWAYS | WP710:DNA damage response only ATM dependent | 16 | 2.50 | 3.19E-05 |
| REACTOME_PATHWAY | R-HSA-449147~Signaling by Interleukins | 38 | 5.95 | 3.40E-05 |
| KEGG_PATHWAY | hsa04919:Thyroid hormone signaling pathway | 16 | 2.50 | 3.65E-05 |
| WIKIPATHWAYS | WP138:Androgen receptor signaling pathway | 14 | 2.19 | 3.68E-05 |
| REACTOME_PATHWAY | R-HSA-5663202~Diseases of signal transduction by growth factor receptors and second messengers | 37 | 5.79 | 3.92E-05 |
| KEGG_PATHWAY | hsa05131:Shigellosis | 24 | 3.76 | 4.00E-05 |
| KEGG_PATHWAY | hsa04659:Th17 cell differentiation | 15 | 2.35 | 4.01E-05 |
| KEGG_PATHWAY | hsa04350:TGF-beta signaling pathway | 15 | 2.35 | 4.01E-05 |
| REACTOME_PATHWAY | R-HSA-9700206~Signaling by ALK in cancer | 14 | 2.19 | 4.41E-05 |
| REACTOME_PATHWAY | R-HSA-9725370~Signaling by ALK fusions and activated point mutants | 14 | 2.19 | 4.41E-05 |
| WIKIPATHWAYS | WP3651:Pathways affected in adenoid cystic carcinoma | 12 | 1.88 | 4.52E-05 |
| WIKIPATHWAYS | WP2446:Retinoblastoma gene in cancer | 14 | 2.19 | 4.70E-05 |
| REACTOME_PATHWAY | R-HSA-1280218~Adaptive Immune System | 53 | 8.29 | 4.78E-05 |
| WIKIPATHWAYS | WP4659:Gastrin signaling pathway | 16 | 2.50 | 4.87E-05 |
| WIKIPATHWAYS | WP5434:Pathways in cancer | 40 | 6.26 | 5.86E-05 |
| KEGG_PATHWAY | hsa01522:Endocrine resistance | 14 | 2.19 | 5.92E-05 |
| WIKIPATHWAYS | WP2374:Oncostatin M signaling pathway | 11 | 1.72 | 6.24E-05 |
| WIKIPATHWAYS | WP5087:Pleural mesothelioma | 36 | 5.63 | 6.46E-05 |
| REACTOME_PATHWAY | R-HSA-9006925~Intracellular signaling by second messengers | 28 | 4.38 | 6.81E-05 |
| REACTOME_PATHWAY | R-HSA-400253~Circadian Clock | 12 | 1.88 | 7.00E-05 |
| WIKIPATHWAYS | WP5205:Clock controlled autophagy in bone metabolism | 13 | 2.03 | 7.04E-05 |
| KEGG_PATHWAY | hsa04668:TNF signaling pathway | 15 | 2.35 | 7.35E-05 |
| REACTOME_PATHWAY | R-HSA-9013424~RHOV GTPase cycle | 9 | 1.41 | 8.23E-05 |
| WIKIPATHWAYS | WP5130:Th17 cell differentiation pathway | 12 | 1.88 | 9.14E-05 |
| KEGG_PATHWAY | hsa04068:FoxO signaling pathway | 16 | 2.50 | 9.23E-05 |
| WIKIPATHWAYS | WP4666:Hepatitis B infection | 18 | 2.82 | 1.03E-04 |
| REACTOME_PATHWAY | R-HSA-199991~Membrane Trafficking | 45 | 7.04 | 1.10E-04 |
| REACTOME_PATHWAY | R-HSA-1257604~PIP3 activates AKT signaling | 25 | 3.91 | 1.12E-04 |
| KEGG_PATHWAY | hsa05417:Lipid and atherosclerosis | 21 | 3.29 | 1.30E-04 |
| KEGG_PATHWAY | hsa04380:Osteoclast differentiation | 16 | 2.50 | 1.30E-04 |
| WIKIPATHWAYS | WP4216:Chromosomal and microsatellite instability in colorectal cancer | 12 | 1.88 | 1.35E-04 |
| KEGG_PATHWAY | hsa01521:EGFR tyrosine kinase inhibitor resistance | 12 | 1.88 | 1.41E-04 |
| KEGG_PATHWAY | hsa05162:Measles | 16 | 2.50 | 1.67E-04 |
| REACTOME_PATHWAY | R-HSA-5688426~Deubiquitination | 26 | 4.07 | 2.15E-04 |
| REACTOME_PATHWAY | R-HSA-2559585~Oncogene Induced Senescence | 8 | 1.25 | 2.19E-04 |
| WIKIPATHWAYS | WP2509:Nanoparticle triggered autophagic cell death | 7 | 1.10 | 2.20E-04 |
| REACTOME_PATHWAY | R-HSA-9013409~RHOJ GTPase cycle | 10 | 1.56 | 2.32E-04 |
| WIKIPATHWAYS | WP2324:AGE RAGE pathway | 11 | 1.72 | 2.59E-04 |
| WIKIPATHWAYS | WP5088:Prostaglandin signaling | 8 | 1.25 | 2.65E-04 |
| REACTOME_PATHWAY | R-HSA-9663891~Selective autophagy | 12 | 1.88 | 2.68E-04 |
| WIKIPATHWAYS | WP179:Cell cycle | 15 | 2.35 | 2.89E-04 |
| REACTOME_PATHWAY | R-HSA-9716542~Signaling by Rho GTPases |  |  |  |
| REACTOME_PATHWAY | R-HSA-9012999~RHO GTPase cycle | 34 | 5.32 | 2.96E-04 |
| REACTOME_PATHWAY | R-HSA-9006936~Signaling by TGFB family members | 15 | 2.35 | 3.02E-04 |
| REACTOME_PATHWAY | R-HSA-1280215~Cytokine Signaling in Immune system | 51 | 7.98 | 3.22E-04 |
| WIKIPATHWAYS | WP4549:Fragile X syndrome | 15 | 2.35 | 3.43E-04 |
| KEGG_PATHWAY | hsa05132:Salmonella infection | 22 | 3.44 | 3.46E-04 |
| WIKIPATHWAYS | WP1971:Integrated cancer pathway | 9 | 1.41 | 3.51E-04 |
| KEGG_PATHWAY | hsa04144:Endocytosis | 22 | 3.44 | 3.66E-04 |
| WIKIPATHWAYS | WP5158:Urotensin II mediated signaling pathway | 11 | 1.72 | 3.76E-04 |
| REACTOME_PATHWAY | R-HSA-9006934~Signaling by Receptor Tyrosine Kinases | 38 | 5.95 | 3.77E-04 |
| WIKIPATHWAYS | WP2263:Androgen receptor network in prostate cancer | 14 | 2.19 | 3.81E-04 |
| WIKIPATHWAYS | WP2261:Glioblastoma signaling pathways | 12 | 1.88 | 3.86E-04 |
| KEGG_PATHWAY | hsa04137:Mitophagy - animal | 13 | 2.03 | 3.93E-04 |
| REACTOME_PATHWAY | R-HSA-5653656~Vesicle-mediated transport | 45 | 7.04 | 4.10E-04 |
| KEGG_PATHWAY | hsa04625:C-type lectin receptor signaling pathway | 13 | 2.03 | 4.30E-04 |
| WIKIPATHWAYS | WP2815:Mammary gland development pathway Involution Stage 4 of 4 | 5 | 0.78 | 4.33E-04 |
| WIKIPATHWAYS | WP5426:HDAC6 interactions in the central nervous system | 14 | 2.19 | 4.97E-04 |
| KEGG_PATHWAY | hsa04621:NOD-like receptor signaling pathway | 18 | 2.82 | 5.06E-04 |
| KEGG_PATHWAY | hsa05135:Yersinia infection | 15 | 2.35 | 5.18E-04 |
| REACTOME_PATHWAY | R-HSA-8856828~Clathrin-mediated endocytosis | 16 | 2.50 | 5.19E-04 |
| REACTOME_PATHWAY | R-HSA-9006931~Signaling by Nuclear Receptors | 25 | 3.91 | 5.46E-04 |
| WIKIPATHWAYS | WP5347:IL 26 signaling pathways | 9 | 1.41 | 5.54E-04 |
| REACTOME_PATHWAY | R-HSA-5357801~Programmed Cell Death | 20 | 3.13 | 5.99E-04 |
| KEGG_PATHWAY | hsa04912:GnRH signaling pathway | 12 | 1.88 | 5.99E-04 |
| REACTOME_PATHWAY | R-HSA-194315~Signaling by Rho GTPases | 46 | 7.20 | 6.13E-04 |
| REACTOME_PATHWAY | R-HSA-9824446~Viral Infection Pathways | 52 | 8.14 | 6.27E-04 |
| WIKIPATHWAYS | WP2637:Interleukin 1 IL 1 structural pathway | 9 | 1.41 | 6.40E-04 |
| WIKIPATHWAYS | WP734:Serotonin receptor 4 6 7 and NR3C signaling | 6 | 0.94 | 7.21E-04 |
| KEGG_PATHWAY | hsa04010:MAPK signaling pathway | 24 | 3.76 | 7.44E-04 |
| WIKIPATHWAYS | WP5124:Alzheimer 39 s disease | 23 | 3.60 | 8.09E-04 |
| WIKIPATHWAYS | WP2857:Mesodermal commitment pathway | 16 | 2.50 | 8.10E-04 |
| REACTOME_PATHWAY | R-HSA-9614085~FOXO-mediated transcription | 10 | 1.56 | 8.29E-04 |
| REACTOME_PATHWAY | R-HSA-5689880~Ub-specific processing proteases | 20 | 3.13 | 8.40E-04 |
| WIKIPATHWAYS | WP673:ErbB signaling pathway | 12 | 1.88 | 8.62E-04 |
| WIKIPATHWAYS | WP2828:Bladder cancer | 8 | 1.25 | 9.09E-04 |
| WIKIPATHWAYS | WP2431:Spinal cord injury | 14 | 2.19 | 9.62E-04 |
| KEGG_PATHWAY | hsa05171:Coronavirus disease - COVID-19 | 20 | 3.13 | 9.90E-04 |
| KEGG_PATHWAY | hsa05152:Tuberculosis | 17 | 2.66 | 9.95E-04 |
| REACTOME_PATHWAY | R-HSA-8939211~ESR-mediated signaling | 20 | 3.13 | 0.001001 |
| KEGG_PATHWAY | hsa05130:Pathogenic Escherichia coli infection | 18 | 2.82 | 0.001029 |
| WIKIPATHWAYS | WP4630:Measles virus infection | 15 | 2.35 | 0.00103 |
| WIKIPATHWAYS | WP2059:Alzheimer 39 s disease and miRNA effects | 23 | 3.60 | 0.001038 |
| WIKIPATHWAYS | WP5115:Network map of SARS CoV 2 signaling pathway | 20 | 3.13 | 0.00112 |
| REACTOME_PATHWAY | R-HSA-1655829~Regulation of cholesterol biosynthesis by SREBP (SREBF) | 9 | 1.41 | 0.001154 |
| KEGG_PATHWAY | hsa05210:Colorectal cancer | 11 | 1.72 | 0.001205 |
| KEGG_PATHWAY | hsa05165:Human papillomavirus infection | 25 | 3.91 | 0.001206 |
| WIKIPATHWAYS | WP195:IL 1 signaling pathway | 9 | 1.41 | 0.001241 |
| WIKIPATHWAYS | WP560:TGF beta receptor signaling | 9 | 1.41 | 0.001403 |
| WIKIPATHWAYS | WP4685:Melanoma | 10 | 1.56 | 0.001427 |
| REACTOME_PATHWAY | R-HSA-9679506~SARS-CoV Infections | 30 | 4.69 | 0.001444 |
| REACTOME_PATHWAY | R-HSA-9679191~Potential therapeutics for SARS | 12 | 1.88 | 0.001495 |
| WIKIPATHWAYS | WP707:DNA damage response | 10 | 1.56 | 0.001585 |
| WIKIPATHWAYS | WP2332:Interleukin 11 signaling pathway | 8 | 1.25 | 0.001629 |
| REACTOME_PATHWAY | R-HSA-109581~Apoptosis | 17 | 2.66 | 0.001645 |
| KEGG_PATHWAY | hsa04151:PI3K-Akt signaling pathway | 26 | 4.07 | 0.00166 |
| KEGG_PATHWAY | hsa04071:Sphingolipid signaling pathway | 13 | 2.03 | 0.001663 |
| WIKIPATHWAYS | WP5381:Smith Magenis and Potocki Lupski syndrome copy number variation | 16 | 2.50 | 0.001683 |
| REACTOME_PATHWAY | R-HSA-9675108~Nervous system development | 38 | 5.95 | 0.001749 |
| REACTOME_PATHWAY | R-HSA-3700989~Transcriptional Regulation by TP53 | 27 | 4.23 | 0.001766 |
| WIKIPATHWAYS | WP481:Insulin signaling | 16 | 2.50 | 0.001792 |
| WIKIPATHWAYS | WP712:Estrogen signaling pathway | 6 | 0.94 | 0.00183 |
| REACTOME_PATHWAY | R-HSA-9013406~RHOQ GTPase cycle | 9 | 1.41 | 0.001837 |
| KEGG_PATHWAY | hsa05220:Chronic myeloid leukemia | 10 | 1.56 | 0.001845 |
| KEGG_PATHWAY | hsa04922:Glucagon signaling pathway | 12 | 1.88 | 0.001927 |
| WIKIPATHWAYS | WP4298:Acute viral myocarditis | 11 | 1.72 | 0.001967 |
| KEGG_PATHWAY | hsa04110:Cell cycle | 15 | 2.35 | 0.001969 |
| REACTOME_PATHWAY | R-HSA-936440~Negative regulators of DDX58/IFIH1 signaling | 7 | 1.10 | 0.002017 |
| KEGG_PATHWAY | hsa05222:Small cell lung cancer | 11 | 1.72 | 0.002019 |
| WIKIPATHWAYS | WP2586:Aryl hydrocarbon receptor pathway | 8 | 1.25 | 0.002125 |
| WIKIPATHWAYS | WP236:Adipogenesis | 14 | 2.19 | 0.002151 |
| WIKIPATHWAYS | WP4816:TGF beta receptor signaling in skeletal dysplasias | 9 | 1.41 | 0.002226 |
| KEGG_PATHWAY | hsa04066:HIF-1 signaling pathway | 12 | 1.88 | 0.002236 |
| REACTOME_PATHWAY | R-HSA-6804757~Regulation of TP53 Degradation | 7 | 1.10 | 0.002343 |
| KEGG_PATHWAY | hsa05017:Spinocerebellar ataxia | 14 | 2.19 | 0.00238 |
| WIKIPATHWAYS | WP3931:Embryonic stem cell pluripotency pathways | 13 | 2.03 | 0.002389 |
| WIKIPATHWAYS | WP363:Wnt signaling pathway | 8 | 1.25 | 0.002413 |
| REACTOME_PATHWAY | R-HSA-8986944~Transcriptional Regulation by MECP2 | 9 | 1.41 | 0.002533 |
| REACTOME_PATHWAY | R-HSA-556833~Metabolism of lipids | 46 | 7.20 | 0.002539 |
| REACTOME_PATHWAY | R-HSA-6806003~Regulation of TP53 Expression and Degradation | 7 | 1.10 | 0.002707 |
| REACTOME_PATHWAY | R-HSA-422475~Axon guidance | 36 | 5.63 | 0.00273 |
| WIKIPATHWAYS | WP69:T cell receptor signaling pathway | 10 | 1.56 | 0.00285 |
| KEGG_PATHWAY | hsa05215:Prostate cancer | 11 | 1.72 | 0.002995 |
| REACTOME_PATHWAY | R-HSA-1266738~Developmental Biology | 69 | 10.80 | 0.003081 |
| WIKIPATHWAYS | WP4331:Neovascularization processes | 7 | 1.10 | 0.00316 |
| REACTOME_PATHWAY | R-HSA-8951664~Neddylation | 20 | 3.13 | 0.00323 |
| WIKIPATHWAYS | WP23:B cell receptor signaling pathway | 11 | 1.72 | 0.003544 |
| REACTOME_PATHWAY | R-HSA-2559583~Cellular Senescence | 17 | 2.66 | 0.003692 |
| KEGG_PATHWAY | hsa05211:Renal cell carcinoma | 9 | 1.41 | 0.003735 |
| WIKIPATHWAYS | WP45:G1 to S cell cycle control | 9 | 1.41 | 0.003739 |
| WIKIPATHWAYS | WP3874:Canonical and non canonical TGF B signaling | 5 | 0.78 | 0.003933 |
| REACTOME_PATHWAY | R-HSA-199418~Negative regulation of the PI3K/AKT network | 12 | 1.88 | 0.004263 |
| WIKIPATHWAYS | WP4172:PI3K Akt signaling pathway | 25 | 3.91 | 0.004517 |
| WIKIPATHWAYS | WP5422:IL 19 signaling pathway | 6 | 0.94 | 0.004539 |
| WIKIPATHWAYS | WP2583:T cell receptor and co stimulatory signaling | 6 | 0.94 | 0.004539 |
| REACTOME_PATHWAY | R-HSA-5205647~Mitophagy | 6 | 0.94 | 0.00465 |
| WIKIPATHWAYS | WP706:Sudden infant death syndrome SIDS susceptibility pathways | 15 | 2.35 | 0.004721 |
| WIKIPATHWAYS | WP4658:Small cell lung cancer | 11 | 1.72 | 0.004819 |
| WIKIPATHWAYS | WP382:MAPK signaling pathway | 20 | 3.13 | 0.004832 |
| REACTOME_PATHWAY | R-HSA-109606~Intrinsic Pathway for Apoptosis | 8 | 1.25 | 0.00502 |
| KEGG_PATHWAY | hsa04660:T cell receptor signaling pathway | 12 | 1.88 | 0.005032 |
| REACTOME_PATHWAY | R-HSA-201556~Signaling by ALK | 6 | 0.94 | 0.005407 |
| REACTOME_PATHWAY | R-HSA-114452~Activation of BH3-only proteins | 6 | 0.94 | 0.005407 |
| KEGG_PATHWAY | hsa05235:PD-L1 expression and PD-1 checkpoint pathway in cancer | 10 | 1.56 | 0.005423 |
| REACTOME_PATHWAY | R-HSA-1989781~PPARA activates gene expression | 12 | 1.88 | 0.005547 |
| REACTOME_PATHWAY | R-HSA-597592~Post-translational protein modification | 76 | 11.89 | 0.005677 |
| KEGG_PATHWAY | hsa04370:VEGF signaling pathway | 8 | 1.25 | 0.005765 |
| WIKIPATHWAYS | WP2272:Pathogenic Escherichia coli infection | 8 | 1.25 | 0.005931 |
| WIKIPATHWAYS | WP4941:GPR143 in melanocytes and retinal pigment epithelium cells | 6 | 0.94 | 0.006166 |
| REACTOME_PATHWAY | R-HSA-201451~Signaling by BMP | 6 | 0.94 | 0.006246 |
| REACTOME_PATHWAY | R-HSA-400206~Regulation of lipid metabolism by PPARalpha | 12 | 1.88 | 0.006294 |
| WIKIPATHWAYS | WP3915:Angiopoietin like protein 8 regulatory pathway | 13 | 2.03 | 0.006391 |
| REACTOME_PATHWAY | R-HSA-199992~trans-Golgi Network Vesicle Budding | 9 | 1.41 | 0.006418 |
| KEGG_PATHWAY | hsa04931:Insulin resistance | 11 | 1.72 | 0.006462 |
| KEGG_PATHWAY | hsa04620:Toll-like receptor signaling pathway | 11 | 1.72 | 0.006462 |
| REACTOME_PATHWAY | R-HSA-983169~Class I MHC mediated antigen processing & presentation | 26 | 4.07 | 0.006893 |
| WIKIPATHWAYS | WP2034:Leptin signaling pathway | 9 | 1.41 | 0.007065 |
| WIKIPATHWAYS | WP5146:Lung pathology of COVID 19 | 5 | 0.78 | 0.007285 |
| WIKIPATHWAYS | WP185:Integrin mediated cell adhesion | 11 | 1.72 | 0.007377 |
| REACTOME_PATHWAY | R-HSA-9701898~STAT3 nuclear events downstream of ALK signaling | 4 | 0.63 | 0.007509 |
| WIKIPATHWAYS | WP4577:Neurodegeneration with brain iron accumulation NBIA subtypes pathway | 7 | 1.10 | 0.007596 |
| KEGG_PATHWAY | hsa04657:IL-17 signaling pathway | 10 | 1.56 | 0.007733 |
| KEGG_PATHWAY | hsa05145:Toxoplasmosis | 11 | 1.72 | 0.007808 |
| WIKIPATHWAYS | WP2032:Thyroid stimulating hormone TSH signaling pathway | 8 | 1.25 | 0.007939 |
| KEGG_PATHWAY | hsa04510:Focal adhesion | 16 | 2.50 | 0.008156 |
| WIKIPATHWAYS | WP3893:Development and heterogeneity of the ILC family | 6 | 0.94 | 0.008165 |
| REACTOME_PATHWAY | R-HSA-9022692~Regulation of MECP2 expression and activity | 6 | 0.94 | 0.008191 |
| REACTOME_PATHWAY | R-HSA-389356~CD28 co-stimulation | 6 | 0.94 | 0.008191 |
| WIKIPATHWAYS | WP1530:miRNA regulation of DNA damage response | 9 | 1.41 | 0.008335 |
| REACTOME_PATHWAY | R-HSA-2559580~Oxidative Stress Induced Senescence | 12 | 1.88 | 0.00851 |
| WIKIPATHWAYS | WP3657:Hematopoietic stem cell gene regulation by GABP alpha beta complex | 5 | 0.78 | 0.00872 |
| KEGG_PATHWAY | hsa05226:Gastric cancer | 13 | 2.03 | 0.009019 |
| WIKIPATHWAYS | WP4674:Head and neck squamous cell carcinoma | 9 | 1.41 | 0.00903 |
| REACTOME_PATHWAY | R-HSA-69231~Cyclin D associated events in G1 | 7 | 1.10 | 0.009045 |
| REACTOME_PATHWAY | R-HSA-73762~RNA Polymerase I Transcription Initiation | 7 | 1.10 | 0.009045 |
| REACTOME_PATHWAY | R-HSA-69236~G1 Phase | 7 | 1.10 | 0.009045 |
| REACTOME_PATHWAY | R-HSA-6783783~Interleukin-10 signaling | 7 | 1.10 | 0.009045 |
| REACTOME_PATHWAY | R-HSA-5633007~Regulation of TP53 Activity | 14 | 2.19 | 0.009134 |
| REACTOME_PATHWAY | R-HSA-5205685~PINK1-PRKN Mediated Mitophagy | 5 | 0.78 | 0.009266 |
| REACTOME_PATHWAY | R-HSA-389357~CD28 dependent PI3K/Akt signaling | 5 | 0.78 | 0.009266 |
| WIKIPATHWAYS | WP4874:CAMKK2 pathway | 6 | 0.94 | 0.009315 |
| KEGG_PATHWAY | hsa05022:Pathways of neurodegeneration - multiple diseases | 29 | 4.54 | 0.009766 |
| REACTOME_PATHWAY | R-HSA-69278~Cell Cycle |  |  |  |
| REACTOME_PATHWAY | R-HSA-74160~Gene expression (Transcription) | 78 | 12.21 | 0.009992 |
| REACTOME_PATHWAY | R-HSA-2122947~NOTCH1 Intracellular Domain Regulates Transcription | 7 | 1.10 | 0.01001 |
| WIKIPATHWAYS | WP5083:Neuroinflammation and glutamatergic signaling | 13 | 2.03 | 0.01006 |
| WIKIPATHWAYS | WP4482:Vitamin D in inflammatory diseases | 5 | 0.78 | 0.010329 |
| REACTOME_PATHWAY | R-HSA-170834~Signaling by TGF-beta Receptor Complex | 10 | 1.56 | 0.010354 |
| WIKIPATHWAYS | WP5352:10q11 21q11 23 copy number variation syndrome | 8 | 1.25 | 0.010408 |
| WIKIPATHWAYS | WP4718:Cholesterol metabolism with Bloch and Kandutsch Russell pathways | 7 | 1.10 | 0.010458 |
| WIKIPATHWAYS | WP313:Hepatocyte growth factor receptor signaling | 6 | 0.94 | 0.010573 |
| WIKIPATHWAYS | WP5406:13q12 12 copy number variation | 6 | 0.94 | 0.010573 |
| KEGG_PATHWAY | hsa05164:Influenza A | 14 | 2.19 | 0.010664 |
| REACTOME_PATHWAY | R-HSA-112409~RAF-independent MAPK1/3 activation | 5 | 0.78 | 0.010881 |
| REACTOME_PATHWAY | R-HSA-9658195~Leishmania infection | 14 | 2.19 | 0.011107 |
| REACTOME_PATHWAY | R-HSA-9824443~Parasitic Infection Pathways | 14 | 2.19 | 0.011107 |
| REACTOME_PATHWAY | R-HSA-1640170~Cell Cycle | 40 | 6.26 | 0.011239 |
| REACTOME_PATHWAY | R-HSA-212436~Generic Transcription Pathway | 65 | 10.17 | 0.011265 |
| WIKIPATHWAYS | WP5366:NF1 copy number variation syndrome | 11 | 1.72 | 0.011566 |
| WIKIPATHWAYS | WP2355:Corticotropin releasing hormone signaling pathway | 10 | 1.56 | 0.011688 |
| KEGG_PATHWAY | hsa04210:Apoptosis | 12 | 1.88 | 0.011796 |
| KEGG_PATHWAY | hsa05020:Prion disease | 19 | 2.97 | 0.011918 |
| WIKIPATHWAYS | WP3617:Photodynamic therapy induced NF kB survival signaling | 6 | 0.94 | 0.011942 |
| KEGG_PATHWAY | hsa04915:Estrogen signaling pathway | 12 | 1.88 | 0.012418 |
| KEGG_PATHWAY | hsa04722:Neurotrophin signaling pathway | 11 | 1.72 | 0.01246 |
| REACTOME_PATHWAY | R-HSA-9634638~Estrogen-dependent nuclear events downstream of ESR-membrane signaling | 5 | 0.78 | 0.012667 |
| REACTOME_PATHWAY | R-HSA-453279~Mitotic G1 phase and G1/S transition | 13 | 2.03 | 0.012803 |
| REACTOME_PATHWAY | R-HSA-9018519~Estrogen-dependent gene expression | 13 | 2.03 | 0.012803 |
| KEGG_PATHWAY | hsa04012:ErbB signaling pathway | 9 | 1.41 | 0.012953 |
| KEGG_PATHWAY | hsa04924:Renin secretion | 8 | 1.25 | 0.013344 |
| WIKIPATHWAYS | WP306:Focal adhesion | 16 | 2.50 | 0.013376 |
| REACTOME_PATHWAY | R-HSA-68911~G2 Phase | 3 | 0.47 | 0.0138 |
| REACTOME_PATHWAY | R-HSA-8849473~PTK6 Expression | 3 | 0.47 | 0.0138 |
| KEGG_PATHWAY | hsa04152:AMPK signaling pathway | 11 | 1.72 | 0.013896 |
| WIKIPATHWAYS | WP2018:RANKL RANK signaling pathway | 7 | 1.10 | 0.014012 |
| WIKIPATHWAYS | WP1528:Physiological and pathological hypertrophy of the heart | 5 | 0.78 | 0.014095 |
| WIKIPATHWAYS | WP1539:Angiogenesis | 5 | 0.78 | 0.014095 |
| WIKIPATHWAYS | WP5231:Hippocampal synaptogenesis and neurogenesis | 5 | 0.78 | 0.014095 |
| REACTOME_PATHWAY | R-HSA-4420097~VEGFA-VEGFR2 Pathway | 10 | 1.56 | 0.01425 |
| WIKIPATHWAYS | WP3594:Circadian rhythm genes | 16 | 2.50 | 0.01455 |
| WIKIPATHWAYS | WP2868:TCA cycle nutrient use and invasiveness of ovarian cancer | 3 | 0.47 | 0.014676 |
| WIKIPATHWAYS | WP722:Serotonin HTR1 group and FOS pathway | 6 | 0.94 | 0.01503 |
| REACTOME_PATHWAY | R-HSA-177929~Signaling by EGFR | 7 | 1.10 | 0.015946 |
| WIKIPATHWAYS | WP3872:Regulation of apoptosis by parathyroid hormone related protein | 5 | 0.78 | 0.016264 |
| REACTOME_PATHWAY | R-HSA-73887~Death Receptor Signaling | 13 | 2.03 | 0.016278 |
| KEGG_PATHWAY | hsa04928:Parathyroid hormone synthesis |  |  |  |
| REACTOME_PATHWAY | R-HSA-168249~Innate Immune System | 56 | 8.76 | 0.016351 |
| WIKIPATHWAYS | WP3969:H19 action Rb E2F1 signaling and CDK Beta catenin activity | 4 | 0.63 | 0.016551 |
| KEGG_PATHWAY | hsa05218:Melanoma | 8 | 1.25 | 0.016598 |
| KEGG_PATHWAY | hsa05223:Non-small cell lung cancer | 8 | 1.25 | 0.016598 |
| KEGG_PATHWAY | hsa04550:Signaling pathways regulating pluripotency of stem cells | 12 | 1.88 | 0.016685 |
| KEGG_PATHWAY | hsa04211:Longevity regulating pathway | 9 | 1.41 | 0.01676 |
| REACTOME_PATHWAY | R-HSA-9006927~Signaling by Non-Receptor Tyrosine Kinases | 7 | 1.10 | 0.017373 |
| REACTOME_PATHWAY | R-HSA-9013405~RHOD GTPase cycle | 7 | 1.10 | 0.017373 |
| REACTOME_PATHWAY | R-HSA-8848021~Signaling by PTK6 | 7 | 1.10 | 0.017373 |
| REACTOME_PATHWAY | R-HSA-9013420~RHOU GTPase cycle | 6 | 0.94 | 0.01823 |
| WIKIPATHWAYS | WP3303:RAC1 PAK1 p38 MMP2 pathway | 8 | 1.25 | 0.018271 |
| WIKIPATHWAYS | WP2864:Apoptosis related network due to altered Notch3 in ovarian cancer | 7 | 1.10 | 0.018332 |
| WIKIPATHWAYS | WP5216:Warburg effect modulated by deubiquitinating enzymes and their substrates | 5 | 0.78 | 0.018632 |
| KEGG_PATHWAY | hsa05203:Viral carcinogenesis | 15 | 2.35 | 0.018859 |
| REACTOME_PATHWAY | R-HSA-1538133~G0 and Early G1 | 5 | 0.78 | 0.019106 |
| REACTOME_PATHWAY | R-HSA-2219528~PI3K/AKT Signaling in Cancer | 10 | 1.56 | 0.019136 |
| KEGG_PATHWAY | hsa04024:cAMP signaling pathway | 16 | 2.50 | 0.019505 |
| REACTOME_PATHWAY | R-HSA-6807070~PTEN Regulation | 12 | 1.88 | 0.019792 |
| WIKIPATHWAYS | WP5367:Rubinstein Taybi syndrome 1 | 4 | 0.63 | 0.020088 |
| KEGG_PATHWAY | hsa05214:Glioma | 8 | 1.25 | 0.020377 |
| WIKIPATHWAYS | WP2203:Thymic stromal lymphopoietin TSLP signaling pathway | 6 | 0.94 | 0.020597 |
| WIKIPATHWAYS | WP2526:PDGF pathway | 6 | 0.94 | 0.020597 |
| WIKIPATHWAYS | WP75:Toll like receptor signaling pathway | 10 | 1.56 | 0.021611 |
| KEGG_PATHWAY | hsa05225:Hepatocellular carcinoma | 13 | 2.03 | 0.021646 |
| REACTOME_PATHWAY | R-HSA-1362300~Transcription of E2F targets under negative control by p107 (RBL1) and p130 (RBL2) in complex with HDAC1 | 4 | 0.63 | 0.022086 |
| REACTOME_PATHWAY | R-HSA-2426168~Activation of gene expression by SREBF (SREBP) | 6 | 0.94 | 0.02214 |
| REACTOME_PATHWAY | R-HSA-447115~Interleukin-12 family signaling | 7 | 1.10 | 0.022175 |
| KEGG_PATHWAY | hsa04962:Vasopressin-regulated water reabsorption | 6 | 0.94 | 0.022264 |
| REACTOME_PATHWAY | R-HSA-453274~Mitotic G2-G2/M phases | 15 | 2.35 | 0.022394 |
| REACTOME_PATHWAY | R-HSA-5621481~C-type lectin receptors (CLRs) | 12 | 1.88 | 0.022771 |
| WIKIPATHWAYS | WP1544:MicroRNAs in cardiomyocyte hypertrophy | 10 | 1.56 | 0.02286 |
| KEGG_PATHWAY | hsa05140:Leishmaniasis | 8 | 1.25 | 0.023207 |
| REACTOME_PATHWAY | R-HSA-194138~Signaling by VEGF | 10 | 1.56 | 0.02384 |
| WIKIPATHWAYS | WP5098:T cell activation SARS CoV 2 | 9 | 1.41 | 0.023892 |
| REACTOME_PATHWAY | R-HSA-1980143~Signaling by NOTCH1 | 8 | 1.25 | 0.023902 |
| WIKIPATHWAYS | WP4970:Galanin receptor pathway | 4 | 0.63 | 0.024008 |
| KEGG_PATHWAY | hsa04145:Phagosome | 12 | 1.88 | 0.025025 |
| KEGG_PATHWAY | hsa05170:Human immunodeficiency virus 1 infection | 15 | 2.35 | 0.025346 |
| REACTOME_PATHWAY | R-HSA-2682334~EPH-Ephrin signaling | 9 | 1.41 | 0.025727 |
| REACTOME_PATHWAY | R-HSA-8934593~Regulation of RUNX1 Expression and Activity | 4 | 0.63 | 0.026066 |
| REACTOME_PATHWAY | R-HSA-9661069~Defective binding of RB1 mutants to E2F1 |  |  |  |
| REACTOME_PATHWAY | R-HSA-9617828~FOXO-mediated transcription of cell cycle genes | 4 | 0.63 | 0.026066 |
| REACTOME_PATHWAY | R-HSA-9659787~Aberrant regulation of mitotic G1/S transition in cancer due to RB1 defects | 4 | 0.63 | 0.026066 |
| REACTOME_PATHWAY | R-HSA-9022699~MECP2 regulates neuronal receptors and channels | 4 | 0.63 | 0.026066 |
| WIKIPATHWAYS | WP3869:Cannabinoid receptor signaling | 5 | 0.78 | 0.026966 |
| KEGG_PATHWAY | hsa05010:Alzheimer disease | 23 | 3.60 | 0.027124 |
| WIKIPATHWAYS | WP5198:Inflammatory bowel disease signaling | 6 | 0.94 | 0.027362 |
| WIKIPATHWAYS | WP364:IL6 signaling pathway | 6 | 0.94 | 0.027362 |
| WIKIPATHWAYS | WP3527:Pre implantation embryo | 7 | 1.10 | 0.027413 |
| WIKIPATHWAYS | WP2849:Hematopoietic stem cell differentiation | 7 | 1.10 | 0.027413 |
| REACTOME_PATHWAY | R-HSA-3304349~Loss of Function of SMAD2/3 in Cancer | 3 | 0.47 | 0.027528 |
| KEGG_PATHWAY | hsa04310:Wnt signaling pathway | 13 | 2.03 | 0.027548 |
| WIKIPATHWAYS | WP4655:Cytosolic DNA sensing pathway | 8 | 1.25 | 0.027738 |
| WIKIPATHWAYS | WP2037:Prolactin signaling pathway | 8 | 1.25 | 0.027738 |
| WIKIPATHWAYS | WP5356:Affected pathways in Duchenne muscular dystrophy | 8 | 1.25 | 0.027738 |
| WIKIPATHWAYS | WP4337:ncRNAs involved in STAT3 signaling in hepatocellular carcinoma | 4 | 0.63 | 0.02831 |
| WIKIPATHWAYS | WP3859:TGF beta signaling in thyroid cells for epithelial mesenchymal transition | 4 | 0.63 | 0.02831 |
| REACTOME_PATHWAY | R-HSA-380320~Recruitment of NuMA to mitotic centrosomes | 9 | 1.41 | 0.028807 |
| REACTOME_PATHWAY | R-HSA-9013423~RAC3 GTPase cycle | 9 | 1.41 | 0.028807 |
| REACTOME_PATHWAY | R-HSA-9009391~Extra-nuclear estrogen signaling | 8 | 1.25 | 0.028976 |
| WIKIPATHWAYS | WP4205:MET in type 1 papillary renal cell carcinoma | 7 | 1.10 | 0.029531 |
| WIKIPATHWAYS | WP5085:Vasopressin regulated water reabsorption | 6 | 0.94 | 0.029896 |
| WIKIPATHWAYS | WP4923:Autophagy | 5 | 0.78 | 0.030166 |
| REACTOME_PATHWAY | R-HSA-450282~MAPK targets/ Nuclear events mediated by MAP kinases | 5 | 0.78 | 0.030372 |
| REACTOME_PATHWAY | R-HSA-182971~EGFR downregulation | 5 | 0.78 | 0.030372 |
| REACTOME_PATHWAY | R-HSA-1368082~RORA activates gene expression | 4 | 0.63 | 0.030404 |
| REACTOME_PATHWAY | R-HSA-983168~Antigen processing: Ubiquitination & Proteasome degradation | 20 | 3.13 | 0.031211 |
| REACTOME_PATHWAY | R-HSA-8878159~Transcriptional regulation by RUNX3 | 9 | 1.41 | 0.032133 |
| KEGG_PATHWAY | hsa05321:Inflammatory bowel disease | 7 | 1.10 | 0.032265 |
| WIKIPATHWAYS | WP5300:TROP2 regulatory signaling | 6 | 0.94 | 0.032573 |
| WIKIPATHWAYS | WP2813:Mammary gland development pathway Embryonic development Stage 1 of 4 | 4 | 0.63 | 0.032992 |
| WIKIPATHWAYS | WP4936:Perturbations to host cell autophagy induced by SARS CoV 2 proteins | 4 | 0.63 | 0.032992 |
| KEGG_PATHWAY | hsa04935:Growth hormone synthesis |  |  |  |
| WIKIPATHWAYS | WP4217:Ebola virus infection in host | 11 | 1.72 | 0.033354 |
| REACTOME_PATHWAY | R-HSA-5656169~Termination of translesion DNA synthesis | 5 | 0.78 | 0.033686 |
| WIKIPATHWAYS | WP5072:Modulators of TCR signaling and T cell activation | 7 | 1.10 | 0.034082 |
| REACTOME_PATHWAY | R-HSA-9020591~Interleukin-12 signaling | 6 | 0.94 | 0.034175 |
| KEGG_PATHWAY | hsa05142:Chagas disease | 9 | 1.41 | 0.034634 |
| KEGG_PATHWAY | hsa04914:Progesterone-mediated oocyte maturation | 9 | 1.41 | 0.034634 |
| KEGG_PATHWAY | hsa04217:Necroptosis | 12 | 1.88 | 0.034682 |
| REACTOME_PATHWAY | R-HSA-438064~Post NMDA receptor activation events | 8 | 1.25 | 0.034734 |
| REACTOME_PATHWAY | R-HSA-166520~Signaling by NTRKs | 11 | 1.72 | 0.034849 |
| REACTOME_PATHWAY | R-HSA-1362277~Transcription of E2F targets under negative control by DREAM complex | 4 | 0.63 | 0.035096 |
| WIKIPATHWAYS | WP2038:Microtubule cytoskeleton regulation | 6 | 0.94 | 0.035395 |
| WIKIPATHWAYS | WP4566:Translation inhibitors in chronically activated PDGFRA cells | 6 | 0.94 | 0.035395 |
| WIKIPATHWAYS | WP4538:Regulatory circuits of the STAT3 signaling pathway | 8 | 1.25 | 0.035632 |
| REACTOME_PATHWAY | R-HSA-5620912~Anchoring of the basal body to the plasma membrane | 9 | 1.41 | 0.035713 |
| REACTOME_PATHWAY | R-HSA-3304351~Signaling by TGF-beta Receptor Complex in Cancer | 3 | 0.47 | 0.035777 |
| WIKIPATHWAYS | WP5180:DYRK1A involvement regarding cell proliferation in brain development | 7 | 1.10 | 0.036518 |
| REACTOME_PATHWAY | R-HSA-450294~MAP kinase activation | 7 | 1.10 | 0.036661 |
| REACTOME_PATHWAY | R-HSA-73893~DNA Damage Bypass | 6 | 0.94 | 0.036985 |
| REACTOME_PATHWAY | R-HSA-9707616~Heme signaling | 6 | 0.94 | 0.036985 |
| REACTOME_PATHWAY | R-HSA-6791312~TP53 Regulates Transcription of Cell Cycle Genes | 6 | 0.94 | 0.036985 |
| REACTOME_PATHWAY | R-HSA-9725371~Nuclear events stimulated by ALK signaling in cancer | 5 | 0.78 | 0.037203 |
| WIKIPATHWAYS | WP4149:White fat cell differentiation | 5 | 0.78 | 0.037212 |
| WIKIPATHWAYS | WP2112:IL 17 signaling pathway | 5 | 0.78 | 0.037212 |
| KEGG_PATHWAY | hsa04936:Alcoholic liver disease | 11 | 1.72 | 0.037296 |
| KEGG_PATHWAY | hsa04120:Ubiquitin mediated proteolysis | 11 | 1.72 | 0.037296 |
| WIKIPATHWAYS | WP5440:EPAC1 and PKA reduction of retinal inflammation | 3 | 0.47 | 0.037956 |
| REACTOME_PATHWAY | R-HSA-8957322~Metabolism of steroids | 12 | 1.88 | 0.038003 |
| WIKIPATHWAYS | WP4863:Host pathogen interaction of human coronaviruses autophagy | 4 | 0.63 | 0.03805 |
| WIKIPATHWAYS | WP428:Wnt signaling | 10 | 1.56 | 0.038298 |
| WIKIPATHWAYS | WP4534:Mechanoregulation and pathology of YAP TAZ via Hippo and non Hippo mechanisms | 6 | 0.94 | 0.038364 |
| REACTOME_PATHWAY | R-HSA-168928~DDX58/IFIH1-mediated induction of interferon-alpha/beta | 8 | 1.25 | 0.038966 |
| REACTOME_PATHWAY | R-HSA-380270~Recruitment of mitotic centrosome proteins and complexes | 8 | 1.25 | 0.038966 |
| REACTOME_PATHWAY | R-HSA-380287~Centrosome maturation | 8 | 1.25 | 0.038966 |
| WIKIPATHWAYS | WP5362:TAR syndrome | 7 | 1.10 | 0.039063 |
| REACTOME_PATHWAY | R-HSA-157118~Signaling by NOTCH | 16 | 2.50 | 0.039308 |
| REACTOME_PATHWAY | R-HSA-109582~Hemostasis | 34 | 5.32 | 0.03999 |
| REACTOME_PATHWAY | R-HSA-5625900~RHO GTPases activate CIT | 4 | 0.63 | 0.040138 |
| REACTOME_PATHWAY | R-HSA-5627117~RHO GTPases Activate ROCKs | 4 | 0.63 | 0.040138 |
| REACTOME_PATHWAY | R-HSA-9634815~Transcriptional Regulation by NPAS4 | 5 | 0.78 | 0.040922 |
| WIKIPATHWAYS | WP2817:Mammary gland development pathway Pregnancy and lactation Stage 3 of 4 | 5 | 0.78 | 0.04106 |
| WIKIPATHWAYS | WP3676:BDNF TrkB signaling | 5 | 0.78 | 0.04106 |
| REACTOME_PATHWAY | R-HSA-69202~Cyclin E associated events during G1/S transition | 8 | 1.25 | 0.041203 |
| WIKIPATHWAYS | WP410:Exercise induced circadian regulation | 6 | 0.94 | 0.04148 |
| KEGG_PATHWAY | hsa05031:Amphetamine addiction | 7 | 1.10 | 0.041497 |
| REACTOME_PATHWAY | R-HSA-5607764~CLEC7A (Dectin-1) signaling | 9 | 1.41 | 0.041572 |
| REACTOME_PATHWAY | R-HSA-9820952~Respiratory Syncytial Virus Infection Pathway | 10 | 1.56 | 0.042792 |
| WIKIPATHWAYS | WP205:IL 7 signaling pathway | 4 | 0.63 | 0.04348 |
| KEGG_PATHWAY | hsa04540:Gap junction | 8 | 1.25 | 0.043657 |
| KEGG_PATHWAY | hsa05230:Central carbon metabolism in cancer | 7 | 1.10 | 0.04404 |
| REACTOME_PATHWAY | R-HSA-447041~CHL1 interactions | 3 | 0.47 | 0.044842 |
| REACTOME_PATHWAY | R-HSA-111453~BH3-only proteins associate with and inactivate anti-apoptotic BCL-2 members | 3 | 0.47 | 0.044842 |
| REACTOME_PATHWAY | R-HSA-8943723~Regulation of PTEN mRNA translation | 3 | 0.47 | 0.044842 |
| REACTOME_PATHWAY | R-HSA-112411~MAPK1 (ERK2) activation | 3 | 0.47 | 0.044842 |
| REACTOME_PATHWAY | R-HSA-9687139~Aberrant regulation of mitotic cell cycle due to RB1 defects | 5 | 0.78 | 0.044845 |
| REACTOME_PATHWAY | R-HSA-9833109~Evasion by RSV of host interferon responses | 5 | 0.78 | 0.044845 |
| KEGG_PATHWAY | hsa05224:Breast cancer | 11 | 1.72 | 0.045488 |
| REACTOME_PATHWAY | R-HSA-416572~Sema4D induced cell migration and growth-cone collapse | 4 | 0.63 | 0.045525 |
| REACTOME_PATHWAY | R-HSA-5218921~VEGFR2 mediated cell proliferation | 4 | 0.63 | 0.045525 |
| REACTOME_PATHWAY | R-HSA-69656~Cyclin A:Cdk2-associated events at S phase entry | 8 | 1.25 | 0.045925 |
| REACTOME_PATHWAY | R-HSA-2173793~Transcriptional activity of SMAD2/SMAD3:SMAD4 heterotrimer | 6 | 0.94 | 0.046245 |
| KEGG_PATHWAY | hsa04960:Aldosterone-regulated sodium reabsorption | 5 | 0.78 | 0.046346 |
| WIKIPATHWAYS | WP2380:Brain derived neurotrophic factor BDNF signaling pathway | 11 | 1.72 | 0.047224 |
| WIKIPATHWAYS | WP5039:SARS CoV 2 innate immunity evasion and cell specific immune response | 7 | 1.10 | 0.047364 |
| WIKIPATHWAYS | WP1559:Transcription factors regulate miRNAs related to cardiac hypertrophy | 3 | 0.47 | 0.047534 |
| REACTOME_PATHWAY | R-HSA-73857~RNA Polymerase II Transcription | 66 | 10.33 | 0.047862 |
| WIKIPATHWAYS | WP395:IL 4 signaling pathway | 6 | 0.94 | 0.04816 |
| WIKIPATHWAYS | WP5373:Osteoarthritic chondrocyte hypertrophy | 6 | 0.94 | 0.04816 |
| WIKIPATHWAYS | WP286:IL 3 signaling pathway | 6 | 0.94 | 0.04816 |
| REACTOME_PATHWAY | R-HSA-2173796~SMAD2/SMAD3:SMAD4 heterotrimer regulates transcription | 5 | 0.78 | 0.048971 |
| KEGG_PATHWAY | hsa04926:Relaxin signaling pathway | 10 | 1.56 | 0.049138 |
| WIKIPATHWAYS | WP4300:Extracellular vesicles in the crosstalk of cardiac cells | 4 | 0.63 | 0.049273 |
| REACTOME_PATHWAY | R-HSA-392499~Metabolism of proteins | 95 | 14.87 | 0.049495 |
| REACTOME_PATHWAY | R-HSA-75153~Apoptotic execution phase | 6 | 0.94 | 0.049611 |
| REACTOME_PATHWAY | R-HSA-6811442~Intra-Golgi and retrograde Golgi-to-ER traffic | 14 | 2.19 | 0.049879 |
